# Understanding the temporal dynamics of estimated environmental niche hypervolumes for marine fishes

**DOI:** 10.1002/ece3.9604

**Published:** 2022-12-13

**Authors:** Daniel Vilas, Robert J. Fletcher, Zachary A. Siders, David Chagaris

**Affiliations:** ^1^ Fisheries and Aquatic Sciences Program, School of Forest, Fisheries, and Geomatics Sciences University of Florida Gainesville Florida USA; ^2^ Nature Coast Biological Station, Institute of Food and Agricultural Sciences University of Florida Cedar Key Florida USA; ^3^ Department of Wildlife Ecology and Conservation University of Florida Gainesville Florida USA

**Keywords:** ecological niche model, Gulf of Mexico, hypervolume, niche shift

## Abstract

Understanding how species respond to the environment is essential in ecology, evolution, and conservation. Abiotic factors can influence species responses and the multi‐dimensional space of abiotic factors that allows a species to grow represents the environmental niche. While niches are often assumed to be constant and robust, they are most likely changing over time and estimation can be influenced by population biology, sampling intensity, and computation methodology. Here, we used a 12‐year time series of survey data to fit annual ecological niche models (ENMs) for 10 marine fish species by using two regression and two machine learning algorithms to evaluate the variation and differentiation of environmental niches. Fitted ENMs were used to develop multi‐dimensional annual and pooled hypervolumes that were evaluated over time and across ENM algorithms, species, and years by computing volume, distance, and dissimilarity metrics for each annual estimated niche. We then investigated potential drivers of estimated hypervolume dynamics including species abundance, species occurrence, sampling effort, salinity, red tides severity, and algorithm. Overall, our results revealed that estimated niches varied over time and across ENM, species, and algorithms. Niche estimation was influenced over time by multiple factors suggesting high complexity on niche dynamics interpretation. Species with high occurrence tended to have a closer representation of the pooled niche and years with higher abundance tended to produce niche expansion. ENM algorithm, sampling effort, seawater salinity, and red tides explained the deviations from the pooled niche. Greater sampling effort led to more comprehensive and complete estimates of species niches. High red tides severity triggered niche contraction. Our results emphasize the predictable effects of population, sampling, and environment on species niche estimation and interpretation, and that each should be considered when performing and interpreting ecological niche analyses. Our niche analysis approach may contribute to effectively quantifying and assessing niche dynamics.

## Graphical abstract

Understanding how species respond to the environment is essential in ecology and conservation. We used niche modeling approaches to quantify and track environmental niche space for marine fish species. Our results emphasize the relevant effect of modeling, population, sampling, and environmental factors on species niche estimation and interpretation over time.

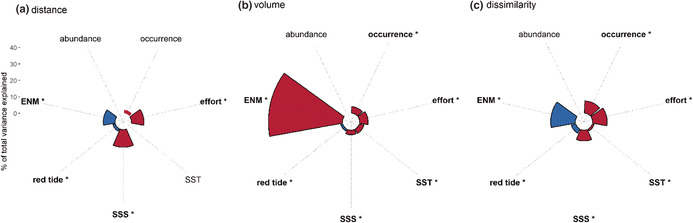

## 1 INTRODUCTION

The spatiotemporal distributions of species are key aspects for addressing issues related to ecology, evolution, and conservation (Elith & Leathwick, 2009). At large spatiotemporal scales, species are distributed over space and time because of the influence of abiotic or environmental factors that affect physiology, phenology, behavior, and geographic ranges. Changes in environmental factors, such as an increase in seawater temperature, may cause shifts in marine species distributions, thus affecting the structure and functioning of marine ecosystems (Pinsky et al., 2020), as well as the ecosystem services they provide (Plagányi, 2019). Therefore, evaluating changes in environmental niche space over time is essential for the conservation and management of species and ecosystems.

Ecological niche theory emphasizes that species use specific resources, habitats, and environments, and their distributions are often based on non‐linear relationships with respect to abiotic factors (Hutchinson, 1957). To describe the ecological niche of a species, Hutchinson (1957) proposed the n‐dimensional hypervolume defined as a multi‐dimensional space of abiotic factors, also known as environmental space, that corresponds to an environmental state that would allow a species to grow and reproduce. The niche conservationism hypothesis states that species and taxonomic groups tend to retain their niches and related ecological traits over space and time (Peterson, 2011; Wiens & Graham, 2005). In practice, recent developments in niche quantification tools can help to better understand the interpretation and estimation of ecological niches over time (Blonder et al., 2017).

Niche estimation could vary over time depending on intrinsic population, environmental, sampling, or temporal scale factors. Population size and competition or predation variation may cause species range contraction or expansion that could alter the estimated ecological niche (Jankowski et al., 2013; von Takach et al., 2020). Environmental perturbations can decrease habitat quality, and cause individuals to move away from impacted areas and therefore temporally affect ecological niche estimation (Fredston et al., 2021; Gannon et al., 2009). Factors related to the sampling process may also influence ecological niche interpretation (Boria et al., 2014). For instance, the extent of the study area can be crucial for species distributions and ecological niche modeling (Barve et al., 2011). At larger temporal scales, ecological niche estimation can be impacted by non‐stationarity in species' environmental relationships (Kingsbury et al., 2020). This process is also known as niche adaptation and may result in a niche expansion. Besides temporal changes, niche estimation can be affected by the algorithm that is applied to predict species occurrence. Several studies evaluated the differences among ecological niche model (ENM) formulation, parametrization, and algorithm predictions (Bucklin et al., 2015; Citores et al., 2020; Norberg et al., 2019), although the effect of ENM algorithm transferability, selection, and parametrization of hypervolume niche estimation remains poorly known.

Species niche variations over time call for developing and implementing methods that quantify and evaluate temporal niche fluctuations. Temporal niche changes may be estimated by using diel activity patterns (e.g., Watabe et al., 2022), diet information (e.g., Grüss et al., 2020), or abundance, biomass, or occurrence information (e.g., von Takach et al., 2020). Multiple tools and analyses have been used to quantify temporal niche differentiation, for example, niche breadth (e.g., White et al., 2015), Principal Component Analysis (e.g., Broennimann et al., 2012), species distribution overlap (e.g., Banerjee et al., 2019), and niche hypervolume (e.g., Carvalho & Cardoso, 2020). Among these methods, multidimensional niche estimation represents a novel and powerful tool to analyze and fully explore niche dynamics over time.

Multiple approaches for estimating species’ ecological niches have since been developed (Sexton et al., 2017). For example, association‐based techniques, like the n‐dimensional hypervolume framework, measure niche breadth through multivariate statistical assessments of species occurrences related to specific abiotic factors (e.g., Blonder et al., 2014). The hypervolume approach quantifies the environmental niche or space that is occupied by a species and represented by the major traits and/or environmental factors affecting a species (Blonder et al., 2014, 2017). The n‐dimensional hypervolume approach provides simple means for comparing niche hypervolumes, and is a powerful tool to assess differences and similarities among ecological niches (Mammola, 2019). Hypervolumes have been successfully applied to quantify, compare and represent realized niches of multiple species and communities and to address ecological, evolutionary, palaeoecological, conservation, and climate change questions (Blonder et al., 2017). For instance, loss of ecological trait diversity was predicted for terrestrial mammals and birds by investigating hypervolumes under future climatic scenarios (Cooke et al., 2019). Additionally, the hypervolume approach can be used to calculate environmental niches by using ENMs and/or species distribution models (SDMs; Blonder et al., 2014; Drake, 2015). Here, we used ENM and SDM terms interchangeably because these terms are closely related (but see Peterson & Soberón, 2012). Some ENM algorithms have been developed beyond the hypervolume concept by including more complexity and biotic and other factors (e.g., Thorson, 2019). Nonetheless, hypervolume approaches can provide insights into the assumptions and behavior of many ENM tools (Blonder et al., 2017).

We addressed the problem of estimating environmental niche hypervolumes over time using a large‐scale marine fish monitoring program spanning a 12‐year period. First, we asked whether the estimation of niche hypervolumes varied over time. To do so, we fitted pooled (12 years of data combined) and year‐specific ENMs for 10 dominant fish taxa across the West Florida Shelf using four modeling algorithms. For each algorithm, we estimated hypervolumes and how components of annual hypervolumes that changed over time related to the 12‐year pooled hypervolume to quantify the extent of temporal dynamics in estimated hypervolumes. Second, we asked what factors may explain variation in hypervolumes over time, focusing on variation in sampling, modeling algorithm used, intrinsic factors that may influence density‐dependent habitat selection, and temporal changes in environmental factors that may alter species responses. Sampling and model algorithms focus on the sensitivity of study design and analysis to affect hypervolume estimation, whereas intrinsic factors and environmental factors focus on how hypothesized processes may expand or constrict the expected hypervolume of a species. This approach may help to determine temporal shifts of niche hypervolumes, explore potential factors affecting the niche temporal interpretation, and find the most appropriate ENM algorithm to capture the responsiveness of species to environmental variation or niche plasticity (Gabriel et al., 2005). The novel framework we present here may help to analyze niche dynamics over time and so disentangle the effects of factors affecting the ecological niche interpretation and estimation over time that could be relevant for management and conservation purposes and improve spatiotemporal ecosystem modeling by accounting for such variability.

## 2 MATERIALS AND METHODS

### 2.1 Study area

**FIGURE 1:**
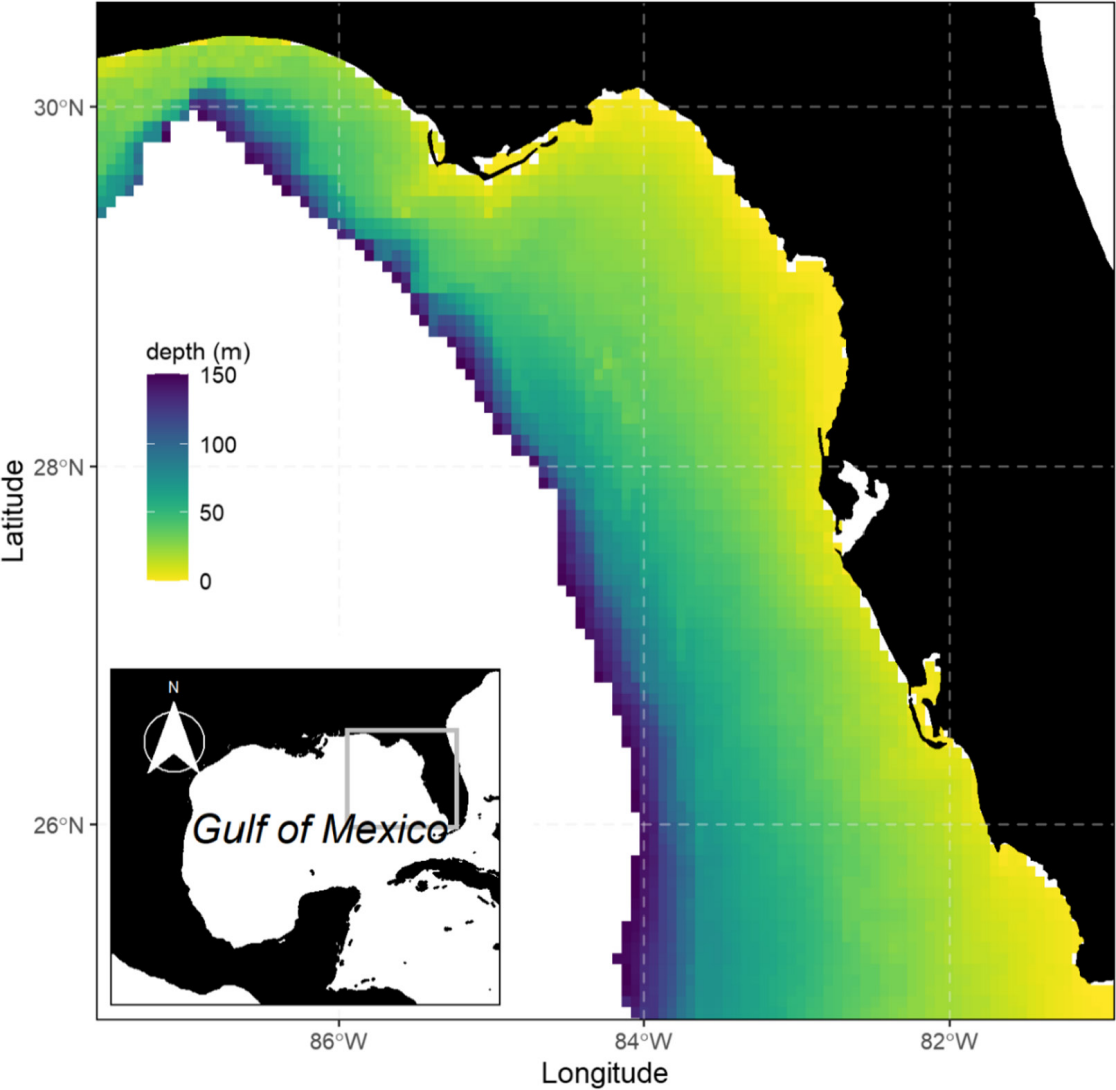
Location of the West Florida shelf study region.

The West Florida Shelf (WFS) is located in the eastern region of the Gulf of Mexico and extends from the west coast of Florida to about 87.5W longitude. The WFS is a broad shelf and covers approximately 170,000 km^2^, with a depth range between 0 and 200 m (Okey & Mahmoudi, 2002; Figure 1). In this region, sea surface temperature fluctuates seasonally between 17°C in winter and 30°C in summer (Liu & Weisberg, 2012), while salinity varies between 5 and 35 parts per thousand, with salinity patterns mainly driven by river outflows. The WFS exhibits high species richness relative to other Gulf of Mexico regions (Harte Research Institute for Gulf of Mexico Studies, 2016; Murawski et al., 2018), fosters a diversity of benthic habitat types, including natural reefs (Darnell, 2015), and supports valuable recreational and commercial fisheries (Florida Fish and Wildlife Conservation Commission, 2020a; NOAA, 2020). However, the WFS region has been impacted by multiple stressors during the last decade: overexploitation, invasive species, red tides, hypoxic events, and oils spill have caused major impacts at ecological levels affecting species, ecosystems, and services (Chagaris et al., 2020; Driggers et al., 2016; Murawski et al., 2016).

### 2.2 Presence–absence data

Presence and absence records were extracted from the Southeast Area Monitoring and Assessment Program (SEAMAP; https://www.gsmfc.org/seamap.php; Rester, 2017) bottom trawl dataset, which is a fishery‐independent survey program operating during summer and fall in the Gulf of Mexico since 1981 and on the WFS since 2008 (Figure S1). During each summer or fall survey, stations were randomly distributed each year on the continental shelf (depth range between 10 and 200 m) and for each station, a 30‐minute tow was conducted with a 40‐ft trawl towed at a speed of 2.5 knots (Figure S2). At each station, all marine organisms were identified, and abundance and biomass were calculated for each species. The SEAMAP trawl survey used a bottom trawl that mainly targets demersal and benthic species.

We filtered the trawl dataset for WFS stations (all since 2009) using latitude and longitude. Then, we selected the 10 most common demersal species in terms of numeric abundance with at least 20 presence points in each year to avoid unreliable predictions since sample size can dictate the ability to capture the environmental responses (Elith & Franklin, 2013; Wood, 2017). The 10 selected demersal species included: Scrawled cowfish (*Acanthostracion quadricornis*), Twospot flounder (*Bothus robinsi*), Littlehead porgy (*Calamus proridens*), Sand perch (*Diplectrum formosum*), Tomtate grunt (*Haemulon aurolineatum*), Pinfish (*Lagodon rhomboides*), Lane snapper (*Lutjanus synagris*), Bluespotted searobin (*Prionotus roseus*), Inshore lizardfish (*Synodus foetens*), and Snakefish (*Trachinocephalus myops*; Table 1). These species capture a broad range of taxa and life‐history strategies and represent the bulk of demersal fish biomass on the WFS dataset (>40% of total fish biomass).

**TABLE 1:** Annual species occurrence of the 10 demersal fish species selected among SEAMAP trawl samples to estimate environmental niche hypervolumes.

Species	2009 (241)	2010 (243)	2011 (151)	2012 (237)	2013 (209)	2014 (297)	2015 (261)	2016 (215)	2017 (257)	2018 (260)	2019 (237)	2020 (90)
*Diplectrum formosum*	199	167	113	188	167	240	215	184	227	215	208	71
*Synodus foetens*	192	187	110	165	148	216	156	154	199	199	187	63
*Acanthostracion quadricornis*	157	102	78	102	130	182	128	121	159	161	147	56
*Haemulon aurolineatum*	163	137	88	163	105	158	139	100	148	135	125	41
*Trachinocephaus myops*	125	126	66	92	120	144	119	108	167	154	148	65
*Lutjanus synagris*	108	82	55	109	90	138	112	89	114	123	97	49
*Bothus robinsi*	96	85	50	66	83	115	103	102	129	118	118	54
*Calamus proridens*	106	115	53	114	82	125	111	80	105	97	83	43
*Prionotus roseus*	91	80	46	71	73	97	97	87	96	111	109	50
*Lagodon rhomboides*	110	68	59	107	67	103	72	41	89	78	78	37

*Note*: Numbers in parentheses represent the number of samples for each year, while numbers in the table indicate the number of samples a given species was observed in the trawl catch.

### 2.3 Environmental data

We used in situ environmental data to fit ENMs and annual environmental raster data to build hypervolumes for comparison purposes. Most stations in the SEAMAP dataset incorporated in situ sea surface temperature (SST), sea surface salinity (SSS), and depth measurements made with conductivity, temperature, and depth (CTD) sensors. To calculate annual environmental data, environmental monthly raster data were extracted from the Hybrid Coordinate Ocean Model (HYCOM; Chassignet et al., 2007) except for depth (NOAA National Geophysical Data Center, 2001). SST and SSS monthly raster data were averaged for each year to compute annual raster data. The spatial resolution of raster data was 0.041 × 0.041 degrees (~5 km). We used environmental raster data for prediction instead of in situ environmental data to ensure that all stations included environmental data. Environmental raster data were scaled by *Z*‐transformation.

### 2.4 Ecological niche models

Species presence–absence was used to fit binomial ecological niche models. Depth, SST, and SSS were considered as potential explanatory variables because of their known influence on the distribution of the marine community (Melo‐Merino et al., 2020). We calibrated annual ENMs and a 12‐year ENM (pooled ENM) for each species in order to evaluate how ENMs varied over time compared to the long‐term (12‐year) average ENM. Modeling each year separately allowed to more clearly interpret temporal variation on the environmental niche. For each species, we fitted annual ENMs by applying two regression algorithms (generalized linear models and generalized additive models) and two machine learning algorithms (random forest and boosted regression trees). Generalized linear models (GLMs) are linear regression models that are based on an assumed relationship using a link function between the response variable and the linear combination of the explanatory variables (Dobson & Barnett, 2018). GLMs can predict non‐linear response functions by adding quadratic or polynomic terms. GLMs were implemented with the *glm* function in [R] (R Core Team, 2017). Generalized additive models (GAMs) are also regression models based on relationships between response and explanatory variables in which smooth functions are additive and provide a flexible method for identifying nonlinear covariate effects (Wood, 2017). GAMs were fitted using the “*mgcv*” package (Wood & Wood, 2015) implemented in [R]. Random forest (RF) models build decision trees using different bootstrap samples of data that predict nonlinear response functions. Decision trees are selected by the bagging method that each occurrence has an equal probability of being selected in subsequent subsamples. Each node is split using the best among a subset of predictors randomly chosen at that node (Breiman, 2001). RF models were fitted using the “*randomForest*” package (Liaw & Wiener, 2002) implemented in [R]. Boosted regression trees (BRT) models combine regression trees with boosting algorithms. Similar to RF, BRT models repeatedly fit many decision trees to improve the accuracy of the model, but BRT uses a boosting method for building decision trees in which occurrence data are weighted in subsequent trees (Elith et al., 2008). BRT models were fitted using the “*gbm*” package (Ridgeway, 2007) implemented in [R].

Selected algorithms were utilized because of their importance in predicting response functions and describing environment niches (Elith & Leathwick, 2009). Each algorithm has some advantages, assumptions, and limitations and may provide different environmental responses (e.g., Norberg et al., 2019). Thus, model selection was not included in the calibration process. For comparison purposes, ENMs were built in the most consistent, parsimonious, and least complex structure possible as we aimed to reproduce similar and flexible response functions. All ENMs were fitted by using binomial information (presence–absence) and formulated to capture nonlinear responses. For GLMs, we added a quadratic term to each explanatory variable so response functions can reproduce unimodal response functions. For GAMs, we implemented the generalized cross‐validation method to estimate smoothing parameters. Additionally, we restricted the degrees of freedom of smooth functions for each explanatory variable (*k* = 4) to avoid oversmoothing and specified a gamma parameter of 1.4 to avoid overfitting (Wood, 2017). RFs and BRTs automatically fit nonlinear response functions through regression trees (Elith et al., 2008), three variables were chosen at each node, and the number of trees was set to as default values (500 and 100 trees, respectively) following previous studies (e.g., Aguirre‐Gutiérrez et al., 2013).

To evaluate fitted ENMs, we used an approach adopted in previous marine species distribution modeling studies (e.g., Grüss et al., 2014, 2021). For each dataset, we used bootstrapping by resampling with replacement (*n* = 1000 bootstrap datasets) to evaluate models. We used this bootstrap method rather than a cross‐validation (leave‐one‐out) method because of the limited number of samples. Then, we evaluated ENMs by computing the area under the curve (AUC), the true skill statistic (TSS), and the root‐mean squared error (RMSE). AUC values, which were obtained by using the ‘*ROCR*’ [R] package (Sing et al., 2005), range from 0 to 1, with 0.5 being as good as random and values close to 1 indicative of perfect prediction (Fielding & Bell, 1997). The TSS index ranges from −1 to +1, where +1 indicates perfect agreement and values of zero or less indicate a performance no better than random (Allouche et al., 2006). The RMSE measured the average prediction error and so the lower the RMSE, the better the model performance.

### 2.5 Hypervolumes

After ENM calibration for each species, models were used to calculate annual three‐dimensional (depth, SST, and SSS) hypervolumes for each species. A complete flowchart of annual hypervolume constructions was represented to visualize the analytical process (Figure S1). The n‐dimensional hypervolume framework was implemented to compute and analyze annual multidimensional hypervolumes for each species by using the “*hypervolume*” [R] package (version 3.04) (Blonder et al., 2014, 2018). Niche hypervolumes are defined by the bounds of scaled and centered environmental factors (Blonder et al., 2018). Specifically, three‐dimensional hypervolumes of species were generated for each year by sampling from fitted ENMs. We used the function *hypervolume_general_model()* to generate hypervolume statistics from an ENM model directly. This approach uses rejection sampling to obtain predicted values of a model object at uniformly random points within a range box from data, then converts the output to a hypervolume and facilitates the interpretation of ENM outputs as hypervolume functions (Blonder et al., 2017).

**FIGURE 2:**
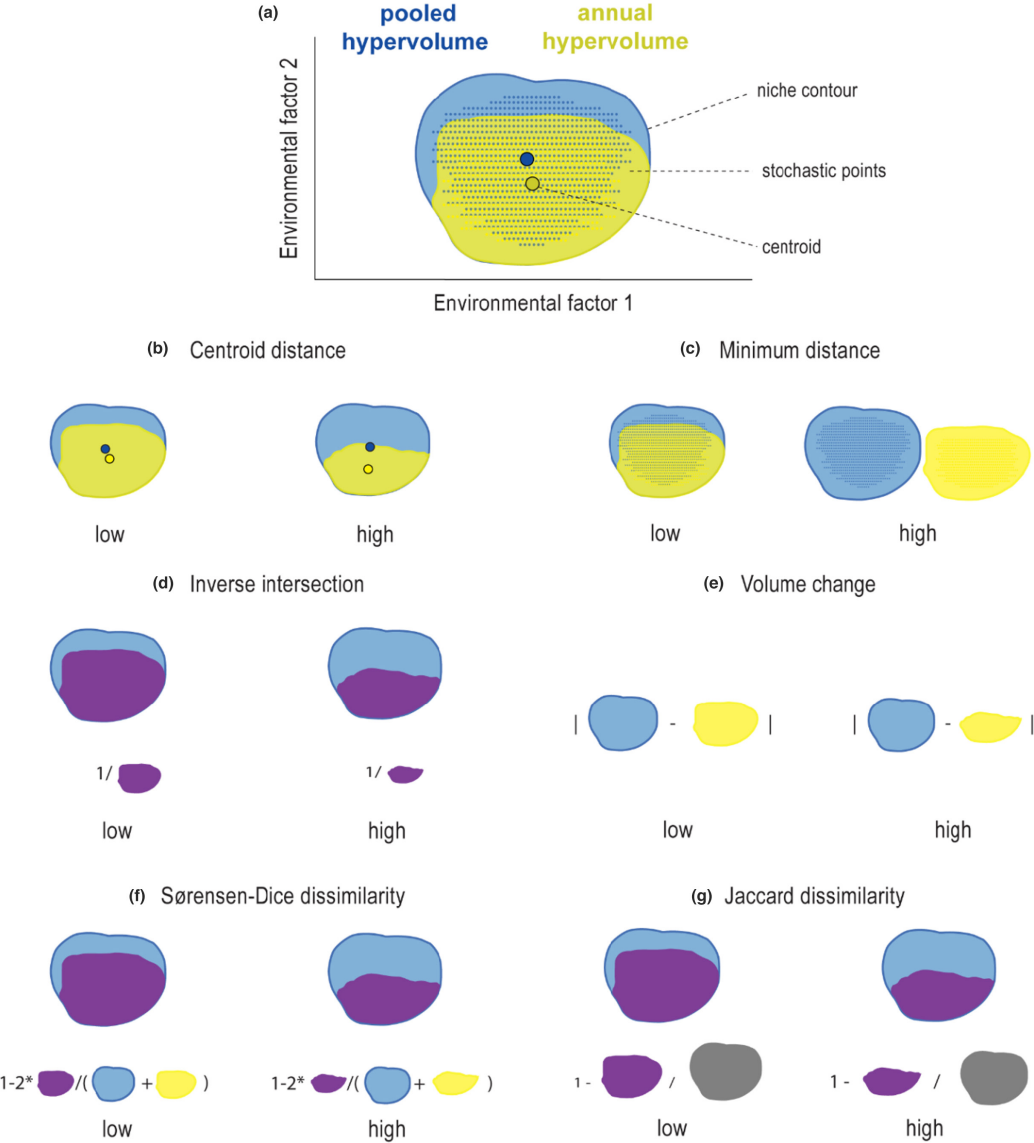
Representation of pooled and annual hypervolumes into a two‐dimensional space defined by two environmental factors and six metrics used to compare hypervolumes (adapted from Mammola, 2019). (a) Each hypervolume represents the environmental niche of a species. Hypervolume contour lines identify and bound environmental niches edges. Stochastic colored points represent the random points that are generated to construct the hypervolumes. Centroids identify the center of the hypervolume. Hypervolumes can be compared using six metrics (b) centroid distance, (c) minimum distance, (d) inverse intersection, (e) volume change, (f) Sørensen‐dice dissimilarity index, and (g) Jaccard dissimilarity index.

To evaluate whether the estimation of niche hypervolumes varied over time, we computed distance, volume, and dissimilarity metrics between each annual hypervolume to the pooled hypervolume for each species (Mammola, 2019; Figure 2a). Such metrics represent a proxy of niche overlap and high niche overlap is expected when the hypervolume comparison obtained low distance, low volume change, low inverse intersection, and low dissimilarity. To evaluate the distance between hypervolumes, we obtained the centroid distance and the minimum distance. The centroid distance is defined as the Euclidean distance between the centroids of two hypervolumes (Figure 2b). The minimum distance represents the pairwise minimum Euclidean distance between two sets of random points comprising two hypervolumes (Figure 2c). To estimate the shared volume between hypervolumes, we used four metrics: the volume change, the inverse intersection, the Sørensen‐Dice dissimilarity index, and the Jaccard dissimilarity index. The inverse intersection of two hypervolumes is calculated as the inverse of the fraction of points falling within the boundaries of both hypervolumes (Figure 2d). The volume change was computed as the absolute volume difference between the pooled and the annual hypervolume (Figure 2e). Both Sørensen‐Dice and Jaccard dissimilarity indices represent the overlap level between two hypervolumes and their values range from 0 (fully overlapped) to 1 (fully disjunct) (Figure 2f,g). Low dissimilarity values indicate a high overlap between the annual estimated niche and the pooled niche.

### 2.6 Linear mixed‐effects models

To explore factors affecting the temporal variation in niche hypervolume estimates, we fitted linear mixed‐effects models (LMMs; Zuur et al., 2009) by using the “*nlme*” [R] package (Pinheiro et al., 2017; version 3.1‐157). We selected centroid distance, volume change, and the Sørensen‐Dice similarity index as response terms because of their variable trend over time and species as a random intercept. For each niche hypervolume metric, we calibrated and evaluated one null model (including only random effects) and seven models that differed in the inclusion of fixed effects that may affect the estimation of niches. LMMs included a temporal autocorrelation term to statistically consider independence on time series estimates.

As fixed effects, we separately investigated multiple factors: two factors representing intrinsic population aspects (annual mean abundance [average caught individuals per year] and occurrence [percentage of stations with at least one individual caught]), one factor related to sampling effort (effort [*n* samples]), three environmental factors (SST, SSS, and red tides severity), and the ENM algorithm. We computed the annual average of temperature and salinity time series from HYCOM data, while the red tides time series was calculated by using *Karenia brevis* cells concentration data collected by the Florida Fish and Wildlife Conservation Commission (FWC; 2020b). Since LMMs differed in their fixed effects structure, we calculated the likelihood‐ratio test (LRT; Harrison et al., 2018) by using the “*lmtest”* [R] package (Hothorn et al., 2015) to compare LMMs. The LRT evaluates the goodness of fit between two models and was used to assess if the inclusion of a fixed effect significantly improves the model fit with respect to the null model. If significant, the variance on the annual niche estimation could be partially explained by each of the considered factors (ANOVA, *p*‐value). For each model containing fixed effects, we also calculated the marginal *R* ^2^ that represents the proportion of variance explained by the fixed effects and the ratio of variance explained by the fixed effects over the total explained variance (Nakagawa & Schielzeth, 2013). The marginal *R* ^2^ comparison helped to elucidate the most important factors affecting temporal niche interpretation.

## 3 RESULTS

Overall, model evaluation results indicated acceptable performance of ENMs. Most ENMs (80%, 416 ENMs out of 520) obtained an AUC value higher than 0.7, a TSS value higher than 0.4, and a RMSE value lower than 0.5 (Table S1). The levels with the highest proportion of inaccurate ENMs (AUC < 0.7, TSS < 0.4, and RMSE > 0.5) were RF for the model algorithm.

**FIGURE 3:**
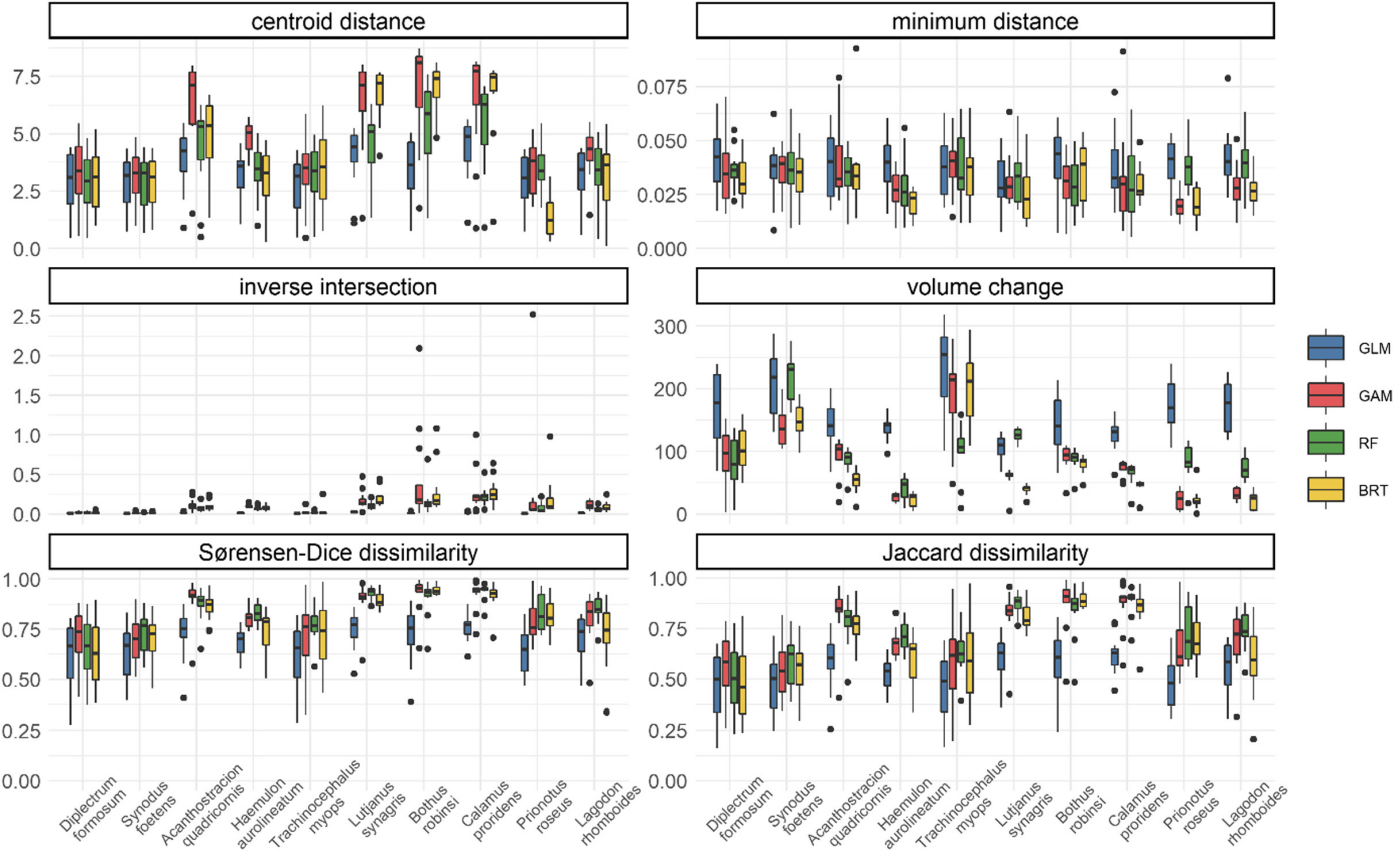
Metric estimates from hypervolume comparison (annual–pooled) across ecological niche modeling (ENM) algorithm and species. Colors represent ENM algorithms: Generalized Linear Model (GLM), Generalized Additive Model (GAM), Random Forest (RF), and Boosted Regression Trees (BRT). Boxplots indicate the distribution over all annual hypervolume values (median, the 10th and the 90th percentiles) for each species and algorithm group. Species are sorted from most to least common.

Species niche results varied among metrics, species, and algorithms (Figure 3; Figure S3). Species and algorithm differences in niche estimations were noticeable for centroid distance, volume change, and dissimilarity metrics (Figure 3). Most annual niche estimates of species were highly dispersed because of the variation over time in these metrics. Regarding species, *Diplectrum formosum* and *Synodus foetens* obtained low centroid distance, inverse intersection, and dissimilarity indicating that low variability in the estimated niche space over time, while other species such as *Lutjanus synagris* showed high values for these metrics (Figure 3). Low distance, inverse intersection and dissimilarity estimates were found for some common species (high occurrence) such as *Diplectrum formosum* (Table 1). In terms of ENM algorithms, the GAM obtained slightly higher centroid distance, while the GLM showed higher volume change. The GAM and RF obtained the highest dissimilarity hypervolume estimates (Figure 3).

**FIGURE 4:**
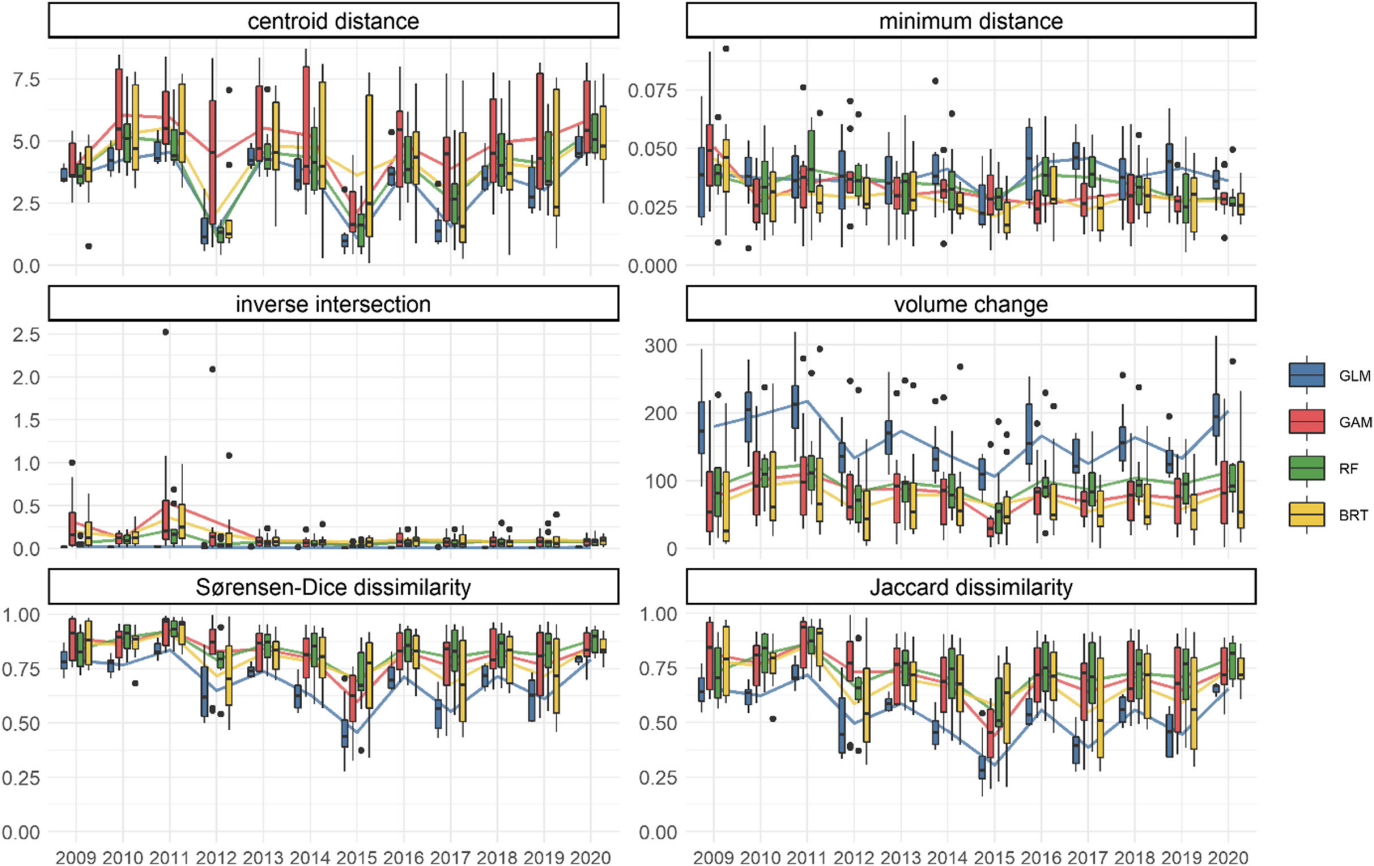
Time series of metric estimates from hypervolume comparison (annual ‐ pooled) across ecological niche modeling (ENM) algorithm. Colors represent ENM algorithms: Generalized Linear Model (GLM), Generalized Additive Model (GAM), Random Forest (RF), and Boosted Regression Trees (BRT). Boxplots indicate distribution over all annual hypervolume values (the median, the 10th, and the 90th percentiles) for each year and algorithm group. Lines indicate mean time series of species across ENM.

Estimated niche dynamics also showed high dispersion, fluctuations, and differences among algorithms, species, and metrics over time (Figure 4; Figure S3). Consistent changes on hypervolume trends over time were captured with centroid distance, volume change, and dissimilarity metrics. Estimated niche dynamics displayed highly dispersed values due to variance among species, especially for the centroid distance and GAM and BRT (Figure 4). Centroid distance, volume change, and dissimilarity metrics fluctuate synchronically and similarly over time. In 2009, 2010, 2013, and 2020, niche estimation obtained high distance, volume change, and dissimilarity indicating low similarity to the pooled niche, while low distance, volume change, and dissimilarity were found in 2012, 2015, 2017, and 2019 (Figure 4).

**FIGURE 5:**
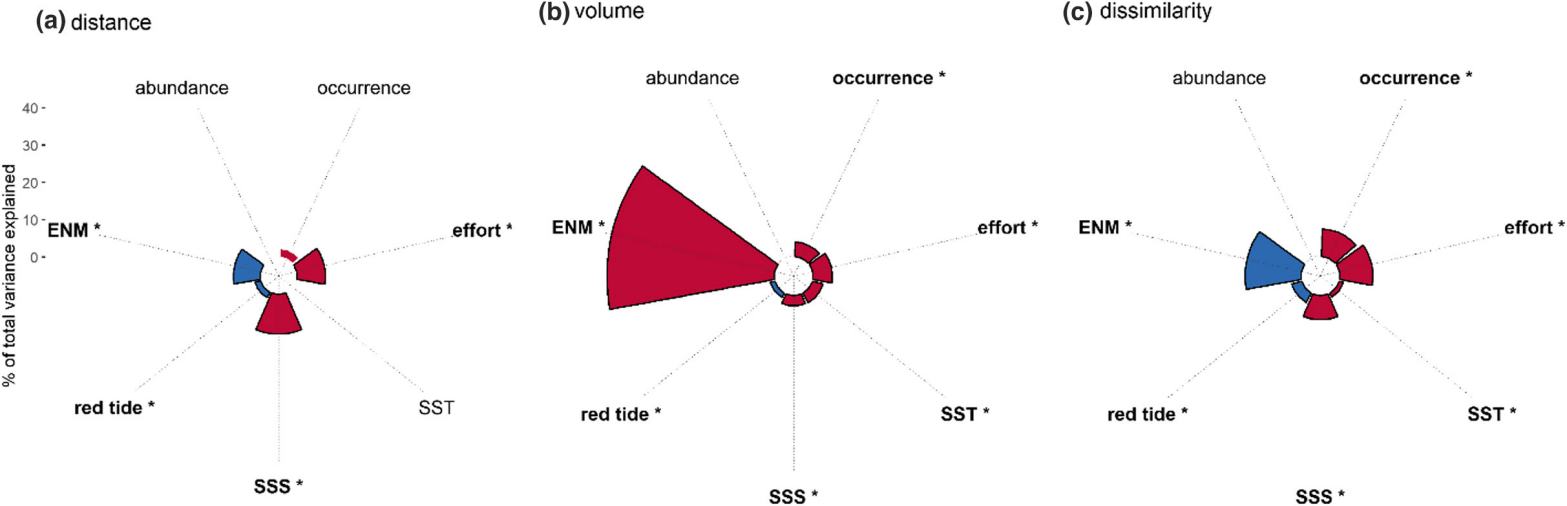
Percent of total variance explained from linear mixed models of each investigated factor for (a) centroid distance, (b) volume change, and (c) Sørensen‐dice dissimilarity index trends. Asterisks and bold sections and factor names identify significant factors (ANOVA, *p* < .05). Blue color represents positive effects and red color represents negative effects.

LMMs results showed that several factors may drive annual niche estimation (Figure 5; Table S2). Volume change obtained the highest variance explained by fixed effects followed by dissimilarity (Figure 5). The most important factors affecting niche temporal estimation were ENM algorithm, occurrence, sampling effort, SSS, and red tides (Table S2). Niche distance dynamics were driven positively by ENM and red tides and negatively by sampling effort, and SSS. Thus, ENM algorithm influenced the niche distance dynamics and high levels of red tides and low of sampling effort, and SSS produced high niche distance. ENM algorithm, occurrence, red tides, sampling effort, SST, and SSS significantly affected the estimation of annual species niche volumes and ENM algorithm explained the highest levels of variance which indicates that volume change was primarily driven by the ENM algorithm choice. Species niche dissimilarity was driven negatively by occurrence, sampling effort, SST, and SSS and positively by ENM algorithm and red tides, which indicates that dissimilarity niche were affected by ENM algorithm and high niche dissimilarity estimates were expected under low occurrence, sampling effort and SSS and high levels of red tides.

## 4 DISCUSSION

Niche quantification is increasingly applied in evolution, conservation, and ecology (e.g., James et al., 2020; Lesser et al., 2020; White et al., 2021). Previous studies demonstrated that estimated niches may contract, expand, or shift over time due to population or environmental factors (e.g., Carvalho & Cardoso, 2020; Chapman et al., 2017; Ern et al., 2017). Our results illustrated that ENM algorithm, environment, sampling effort, and species prevalence may affect the species niche interpretation. This study also demonstrated the capabilities of this methodology to analyze niche dynamics over time. Therefore, such temporal niche approach may be relevant for conservation, management, and species distribution modeling.

### 4.1 The dynamics of niche hypervolumes

Our results indicated that ENM predictions were accurate and temporal niche interpretations were robust. Species displayed differences in terms of mean metric estimates and interannual variability. Interspecific niche distinction is primarily driven by their contrasting preferences for particular resources, habitats, and environments (Sexton et al., 2017). The species results showed that some common species like *Diplectrum formosum* obtained lower mean levels of distance, and dissimilarity among years and so high niche overlap between annual and pooled. These results supported that niche estimation may be affected by population processes such as range contraction or expansion. Species traits and information, for example, species prevalence, can provide conclusions on conservation status, risk assessment, and gear catchability (Chapman et al., 2018; Enquist et al., 2019; Young et al., 2019). Species prevalence may also bias model estimates (Sillero et al., 2021). Common species tend to remain consistent over ecological timescales (Gaston, 2011) and produce a more statistically reliable relationship with environmental factors than least common (Segurado & Araujo, 2004) and least common species tend to be misspecified (Smith et al., 2019). On the other hand, such niche differences across species may be caused by the contrasting plasticity and vulnerability of species to changing environmental conditions (Gabriel et al., 2005; Sexton et al., 2017).

The RF algorithm obtained high dissimilarity hypervolume estimates. Machine learning methods like RF demonstrated strong performance in predicting outcomes (Elith, 2019). High dissimilarity levels may suggest a poor informative estimated niche, but this may help to capture niche fluctuations. These results are in line with the low level of accuracy of the hypervolumes fitted using RF. The high centroid distance and dissimilarity revealed for GAM and the high volume change for GLM may suggest the ability of such ENM algorithm to capture niche fluctuations. This also can indicate more relaxed environmental response curves and niche space estimation that may be uninformative for conservation purposes by overestimating species niche (Warren et al., 2020), but it can represent an advantage for studies with alternative objectives such as coupling ENMs with ecosystem models (Coll et al., 2019). Recent studies recommended GAMs over other approaches for computing environmental response functions (Brodie et al., 2020; Püts et al., 2020). In marine environments, correlative models such as GAM have been more frequently used than other machine learning approaches. However, no single best ecological niche algorithm exists because its predictive power depends on the approach's assumptions and the particularities of the species (Qiao et al., 2015).

### 4.2 Factors explaining temporal variability in hypervolumes

ENM algorithm was identified as an important factor explaining fluctuations in estimated niche distance, volume, and dissimilarity. ENM algorithm should be considered when evaluating niche changes over time, specially niche volume variations, and GLM and GAM may help to better capture such temporal niche variations because of their ability to differentiate among annual niche estimates and the pooled niche estimates. We recommend to explore multiple ENM algorithms when investigating niche fluctuations because ENM algorithms results may differ depending on species traits (Qiao et al., 2015). Occurrence explained a proportion of variance of estimated niche dissimilarity and volume trends. The high occurrence was associated with lower estimated niche dissimilarity and volume, indicating little interannual variation in hypervolume for commonly occurring species. Occurrence and abundance can induce range expansion or contraction in marine species (Thorson et al., 2016; von Takach et al., 2020) and thus alter the temporal niche interpretation. Population size may affect niche distance, position, and volume and thus it should be considered when temporally interpreting species niches. The population size effect could be further explored and reduced if necessary by fitting ENMs with abundance data instead of presence/absence data.

Sampling effort results showed relevant effects for distance, volume, and dissimilarity niche time series and this factor mainly explained niche similarity. Its negative effects for dissimilarity, distance, and volume indicated that the higher the sampling effort, the more comprehensive and complete species niches were obtained. The number of samples is a key aspect of ENMs and may affect the model performance and accuracy (Hernandez et al., 2006; Siders et al., 2020; Stockwell & Peterson, 2002). These results demonstrated that sample design may affect niche interpretation because variation in sampling effort could bias niche estimates.

Our results suggested negative effects of salinity and temperature on niche distance, volume change, and dissimilarity. The increasing seawater salinity and temperature due to climate change may affect species ranges (McHenry et al., 2019; Purtlebaugh et al., 2020) or pushing species to occupy restricted refugia (Stralberg et al., 2020) and consequently altering the ecosystem and the food web. However, the influence of SSS and SST on niche dynamics represents an unexpected finding that may be due to the effect of a small number of samples on nearshore estuarine locations with low salinity levels (Figure S1). This uncertainty could potentially be reduced by incorporating sampling over a broader range of the species' geographic distribution and environmental space. The effect of environment on niche over time indicated that niche estimation could become more uncertain under a climatic change as species distribution is shifting. This might result in ENMs and predictions about species distributions that are obsolete, and it would require routine long‐term monitoring data to account for the effect of changing environmental conditions. Uncertainty may be reduced by increasing the study area to capture a broad range of species geographic distribution and so environmental conditions. By tracking the effect of environmental factors such as SSS on niche estimation, the temporal variation on the niche interpretation may provide comprehensive information on species niche and its conservation under climate change. Other factors such as food availability, water quality, and top‐down predation affect species physiology, distribution, fitness, behavior, phenology, and growth (Alfonso et al., 2021; Free et al., 2019; Pinsky et al., 2013), and should also be investigated to interpret species niches.

Similar to SST and SSS, red tides helped to explain variance in distance, volume, and dissimilarity. Red tides increased niche distance, volume, and dissimilarity, so high levels of red tides could impact estimated niches and hamper its interpretation. These estimated changes may be caused by species range contraction as shown with other stressors (Scheele et al., 2017). In the eastern Gulf of Mexico, periodic red tide events impact fish populations (Sagarese et al., 2021), communities, and ecosystems (Gray DiLeone & Ainsworth, 2019) and may cause emigration from impacted areas (Vilas et al., 2021). The WFS region experienced severe red tide events between 2005 and 2020 (Karnauskas et al., 2019; Walter III et al., 2015) which was captured by the present niche temporal assessment, for instance, the niche dissimilarity in 2019, and 2020. The low proportion of variance explained could be due to the localized and often short‐lived nature of red tides. Severe red tides typically occur close to shore in southwest Florida during the late summer and fall, which rarely coincides with the SEAMAP trawl samples. Despite this, it is possible that the impacts of a red tide bloom span beyond the immediate area and could persist longer than the bloom itself as hypoxic conditions often develop. Understanding how episodic, spatially restricted stressors impact niche interpretation is essential in regions affected by multiple stressors such as the Gulf of Mexico and may be further explored to investigate species vulnerability and plasticity.

### 4.3 Limitations and uncertainties

We demonstrated that the hypervolume approach is a powerful tool to evaluate niche interpretation. Environmental and biological information play important roles in ENMs and hypervolume approaches and more comprehensive data in the marine environment would improve this temporal hypervolume assessment and allow the emergence of new species distribution predictors. Hypervolumes were estimated based on environmental factors with low contrast in the region which may complicate the niche temporal evaluation. This modeling approach followed a standard protocol (Zurell et al., 2020), but we recognize that alternative model structures may alter hypervolume outcomes. Consistent and basic ENM structures were assumed in this study because the model structure could affect hypervolume estimations, but a deeper examination of model structures was beyond the scope of this work. Another limitation faced during this study was that this sample design did not cover the entire geographic distribution of the species, thus we may not fully capture the environmental space of such species, nor population size effects. We acknowledge that accessible area of species is crucial for niche modeling (Barve et al., 2011), but the fully quantification of species‐realized niches requires high computational capacity and a complete sampling coverage. In line with that, some ENMs may not produce bounded niche hypervolumes that may be determined by spatial scale of the study and so the range of values of environmental factors. Although the geographical space was relatively limited in relation to the geographic ranges of these species, this did not preclude the ability to capture changes in the estimated environmental niches among species, years, and ENM algorithms from sampling‐based information at this spatial scale.

## 5 CONCLUSION

Our results demonstrate the effects of ENM algorithm, population, sampling, and environmental factors on species niche estimation and interpretation. Such factors caused fluctuations in species estimated niche and affected the pooled niche estimate. We suggest a preliminary examination of factors affecting the estimated niche dynamics that should be corrected when comparing niche estimates. We should rather calculate a pooled niche for a comprehensive estimate that can remove the temporal fluctuation on the estimated niche and help to provide management and conservation advice. Biased niche estimates may impact species response functions and predicted geographic space that may lead to erroneous management and conservation decisions. Niche interpretation may be relevant for studies in which trophic functions may have a critical influence on spatial ecosystem model dynamics (Plagányi, 2007; Shin et al., 2010) such as Ecospace (Christensen et al., 2008). The study demonstrated that our niche analysis approach may contribute to effectively quantifying and assessing niche dynamics. By evaluating the temporal niche variability, we showed the effect of environmental perturbations on the estimated niche. This may help to understand the resiliency of some species to environmental perturbations and rapid environmental changes that can improve the management and conservation of species, for example, by providing spaces where impact mitigation is possible (Scheele et al., 2017). In the future, stressors such as red tide events are expected to increase in terms of occurrence and magnitude (Anderson et al., 2021). Evaluating changes in temporal niche volume of species may help assess the adaptability, genetic diversity, and evolutionary responses to perturbation events.

## AUTHOR CONTRIBUTIONS

**Daniel Vilas:** Conceptualization (equal); data curation (lead); formal analysis (lead); methodology (equal); visualization (lead); writing – original draft (lead); writing – review and editing (lead). **Robert J. Fletcher Jr.:** Conceptualization (lead); writing – original draft (supporting); writing – review and editing (supporting). **Zachary A. Siders:** Data curation (supporting); formal analysis (supporting); methodology (supporting); writing – review and editing (supporting). **David Chagaris:** Conceptualization (supporting); funding acquisition (lead); writing – original draft (supporting); writing – review and editing (supporting).

## Supporting information


Appendix S1

## Data Availability

The estimated niche data that support the findings of this study are openly available in “figshare” at https://doi.org/10.6084/m9.figshare.20826235.v1.
