# Supplementary material for: Understanding the temporal dynamics of estimated environmental niche hypervolumes for marine fishes
Source: Ecol Evol. 2022 Dec 13;12(12):e9604. doi: 10.1002/ece3.9604 (PMC9748244; doi:10.1002/ece3.9604)
Supplement: Supplementary file 1 — Appendix S1 [file ECE3-12-e9604-s001.docx]

**Understanding the temporal dynamics of estimated environmental niche hypervolumes for marine fishes**

Daniel Vilas^1,2^, Robert J. Fletcher, Jr.^3^, Zachary Siders^1^, David Chagaris^1,2^

^1^ Fisheries and Aquatic Sciences Program, School of Forest, Fisheries, and Geomatics Sciences, University of Florida, Gainesville, FL 32611, United States;

^2^ Nature Coast Biological Station, Institute of Food and Agricultural Sciences, University of Florida, Cedar Key, FL 32625, United States;

^3^ Department of Wildlife Ecology and Conservation, University of Florida, Gainesville, FL, 32611, United States.

**Appendix A**


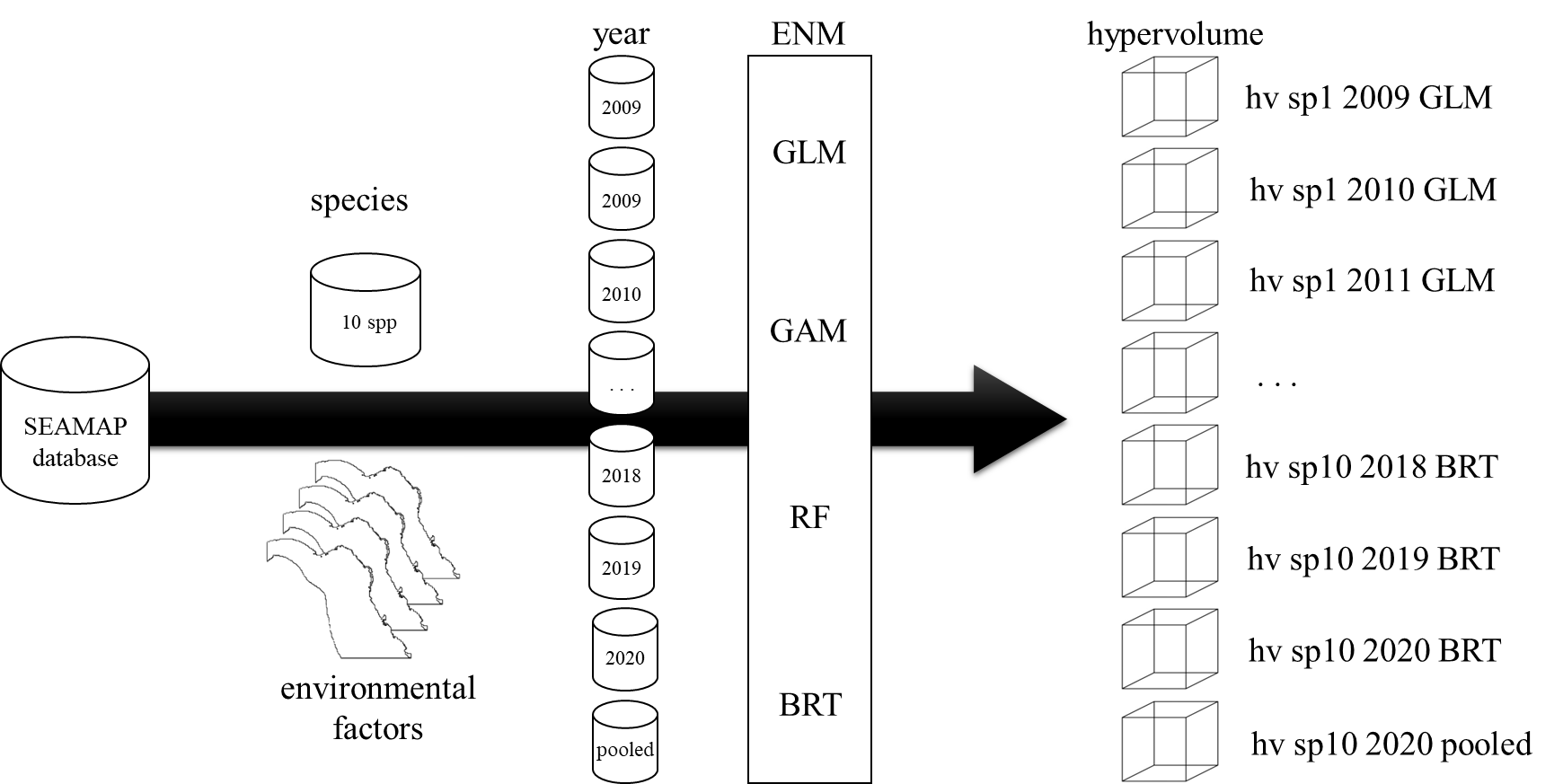


Figure S1. Flowchart of the analytic process to produce multidimensional hypervolumes (hv) for each species (sp), year, pooled years, and ecological niche model (ENM) algorithm. First, annual and pooled binomial ENMs were fitted using SEAMAP trawl data from the WFS. Second, annual and pooled hypervolumes were fitted with predicted probability of presence and environmental HYCOM data. (WFS: West Florida Shelf; SEAMAP: Southeast Area Monitoring and Assessment Program; GLM: Generalized Linear Model; GAM: Generalized Additive.


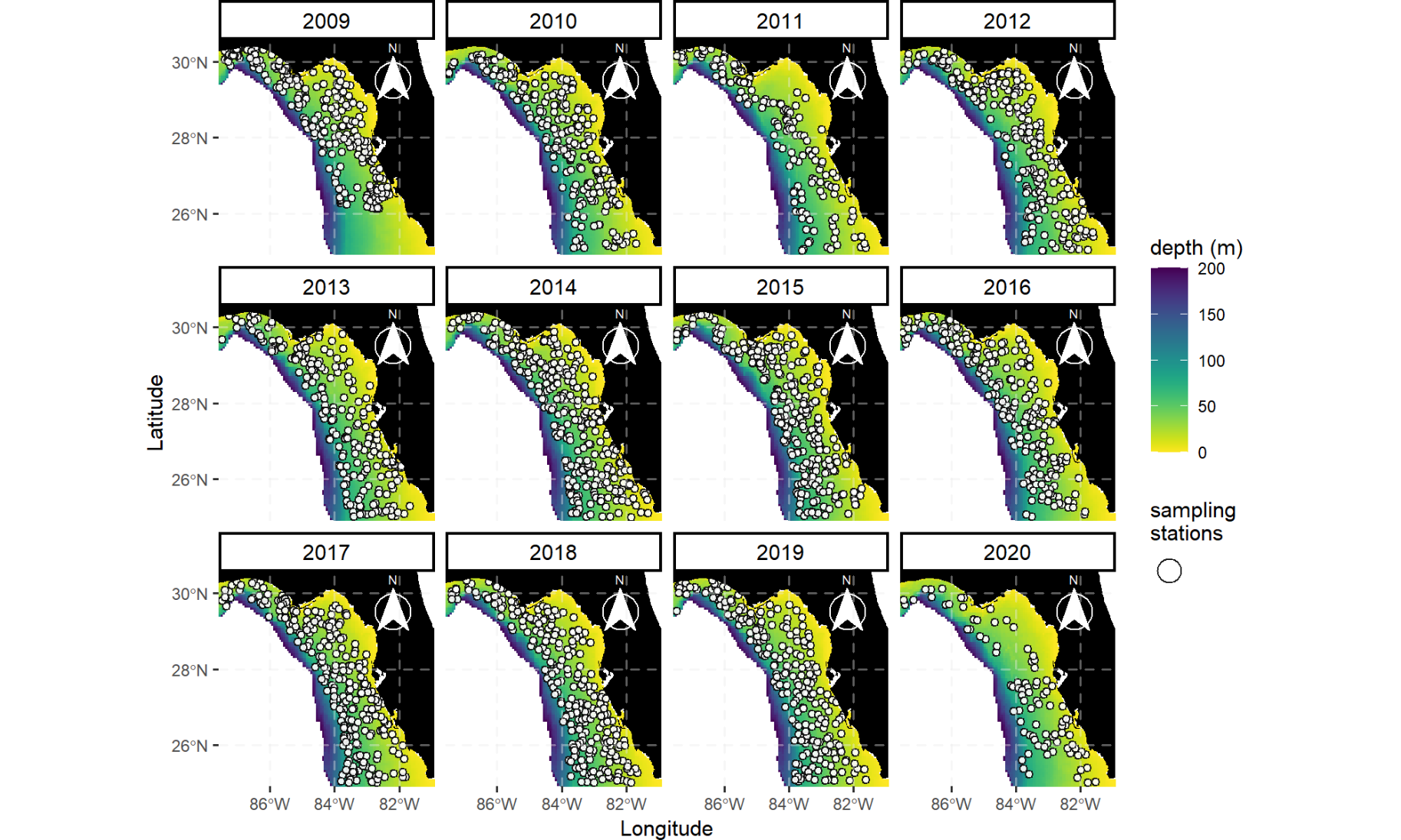


Figure S2. Location of selected SEAMAP trawl sampling stations on the West Florida Shelf region by year.

Table S1. Root mean-square error (RMSE), area under the curve (AUC) and true skill statistic (TSS) value for each species, year, and ecological niche model (ENM) algorithm (GLM: Generalized Linear Model; GAM: Generalized Additive Model; RF: Random Forest; BRT: Boosted Regression Trees) to evaluate ENMs.

| **species** | **year** | **ENM** | **RMSE** | **AUC** | **TSS** |
| --- | --- | --- | --- | --- | --- |
| *Acanthostracion quadricornis* | 2009 | GAM | 0.359 | 0.891 | 0.655 |
| *Acanthostracion quadricornis* | 2009 | GLM | 0.411 | 0.809 | 0.485 |
| *Acanthostracion quadricornis* | 2009 | RF | 0.503 | 0.719 | 0.460 |
| *Acanthostracion quadricornis* | 2009 | BRT | 0.330 | 0.925 | 0.702 |
| *Acanthostracion quadricornis* | 2010 | GAM | 0.357 | 0.898 | 0.662 |
| *Acanthostracion quadricornis* | 2010 | GLM | 0.385 | 0.883 | 0.633 |
| *Acanthostracion quadricornis* | 2010 | RF | 0.470 | 0.767 | 0.533 |
| *Acanthostracion quadricornis* | 2010 | BRT | 0.338 | 0.918 | 0.696 |
| *Acanthostracion quadricornis* | 2011 | GAM | 0.337 | 0.912 | 0.751 |
| *Acanthostracion quadricornis* | 2011 | GLM | 0.355 | 0.907 | 0.672 |
| *Acanthostracion quadricornis* | 2011 | RF | 0.415 | 0.828 | 0.658 |
| *Acanthostracion quadricornis* | 2011 | BRT | 0.310 | 0.941 | 0.805 |
| *Acanthostracion quadricornis* | 2012 | GAM | 0.415 | 0.816 | 0.521 |
| *Acanthostracion quadricornis* | 2012 | GLM | 0.422 | 0.811 | 0.498 |
| *Acanthostracion quadricornis* | 2012 | RF | 0.512 | 0.729 | 0.472 |
| *Acanthostracion quadricornis* | 2012 | BRT | 0.378 | 0.878 | 0.610 |
| *Acanthostracion quadricornis* | 2013 | GAM | 0.402 | 0.814 | 0.501 |
| *Acanthostracion quadricornis* | 2013 | GLM | 0.414 | 0.800 | 0.550 |
| *Acanthostracion quadricornis* | 2013 | RF | 0.513 | 0.711 | 0.435 |
| *Acanthostracion quadricornis* | 2013 | BRT | 0.379 | 0.866 | 0.521 |
| *Acanthostracion quadricornis* | 2014 | GAM | 0.420 | 0.802 | 0.465 |
| *Acanthostracion quadricornis* | 2014 | GLM | 0.422 | 0.800 | 0.454 |
| *Acanthostracion quadricornis* | 2014 | RF | 0.542 | 0.691 | 0.394 |
| *Acanthostracion quadricornis* | 2014 | BRT | 0.402 | 0.842 | 0.523 |
| *Acanthostracion quadricornis* | 2015 | GAM | 0.439 | 0.768 | 0.393 |
| *Acanthostracion quadricornis* | 2015 | GLM | 0.455 | 0.740 | 0.389 |
| *Acanthostracion quadricornis* | 2015 | RF | 0.564 | 0.680 | 0.340 |
| *Acanthostracion quadricornis* | 2015 | BRT | 0.416 | 0.824 | 0.537 |
| *Acanthostracion quadricornis* | 2016 | GAM | 0.467 | 0.701 | 0.334 |
| *Acanthostracion quadricornis* | 2016 | GLM | 0.468 | 0.696 | 0.322 |
| *Acanthostracion quadricornis* | 2016 | RF | 0.632 | 0.601 | 0.208 |
| *Acanthostracion quadricornis* | 2016 | BRT | 0.427 | 0.812 | 0.470 |
| *Acanthostracion quadricornis* | 2017 | GAM | 0.432 | 0.763 | 0.467 |
| *Acanthostracion quadricornis* | 2017 | GLM | 0.438 | 0.758 | 0.337 |
| *Acanthostracion quadricornis* | 2017 | RF | 0.526 | 0.702 | 0.417 |
| *Acanthostracion quadricornis* | 2017 | BRT | 0.390 | 0.853 | 0.525 |
| *Acanthostracion quadricornis* | 2018 | GAM | 0.429 | 0.767 | 0.447 |
| *Acanthostracion quadricornis* | 2018 | GLM | 0.430 | 0.766 | 0.459 |
| *Acanthostracion quadricornis* | 2018 | RF | 0.545 | 0.685 | 0.379 |
| *Acanthostracion quadricornis* | 2018 | BRT | 0.400 | 0.835 | 0.490 |
| *Acanthostracion quadricornis* | 2019 | GAM | 0.452 | 0.710 | 0.306 |
| *Acanthostracion quadricornis* | 2019 | GLM | 0.451 | 0.712 | 0.338 |
| *Acanthostracion quadricornis* | 2019 | RF | 0.559 | 0.668 | 0.361 |
| *Acanthostracion quadricornis* | 2019 | BRT | 0.415 | 0.820 | 0.523 |
| *Acanthostracion quadricornis* | 2020 | GAM | 0.322 | 0.923 | 0.694 |
| *Acanthostracion quadricornis* | 2020 | GLM | 0.354 | 0.912 | 0.744 |
| *Acanthostracion quadricornis* | 2020 | RF | 0.459 | 0.778 | 0.564 |
| *Acanthostracion quadricornis* | 2020 | BRT | 0.308 | 0.944 | 0.768 |
| *Acanthostracion quadricornis* | pooled | GAM | 0.452 | 0.740 | 0.353 |
| *Acanthostracion quadricornis* | pooled | GLM | 0.456 | 0.734 | 0.334 |
| *Acanthostracion quadricornis* | pooled | RF | 0.567 | 0.674 | 0.346 |
| *Acanthostracion quadricornis* | pooled | BRT | 0.449 | 0.750 | 0.378 |
| *Bothus robinsi* | 2009 | GAM | 0.413 | 0.813 | 0.480 |
| *Bothus robinsi* | 2009 | GLM | 0.438 | 0.777 | 0.435 |
| *Bothus robinsi* | 2009 | RF | 0.569 | 0.659 | 0.323 |
| *Bothus robinsi* | 2009 | BRT | 0.394 | 0.852 | 0.525 |
| *Bothus robinsi* | 2010 | GAM | 0.419 | 0.779 | 0.427 |
| *Bothus robinsi* | 2010 | GLM | 0.424 | 0.779 | 0.376 |
| *Bothus robinsi* | 2010 | RF | 0.589 | 0.602 | 0.211 |
| *Bothus robinsi* | 2010 | BRT | 0.393 | 0.841 | 0.525 |
| *Bothus robinsi* | 2011 | GAM | 0.387 | 0.844 | 0.524 |
| *Bothus robinsi* | 2011 | GLM | 0.399 | 0.844 | 0.519 |
| *Bothus robinsi* | 2011 | RF | 0.531 | 0.680 | 0.358 |
| *Bothus robinsi* | 2011 | BRT | 0.350 | 0.906 | 0.602 |
| *Bothus robinsi* | 2012 | GAM | 0.419 | 0.710 | 0.248 |
| *Bothus robinsi* | 2012 | GLM | 0.418 | 0.720 | 0.278 |
| *Bothus robinsi* | 2012 | RF | 0.545 | 0.574 | 0.205 |
| *Bothus robinsi* | 2012 | BRT | 0.387 | 0.806 | 0.403 |
| *Bothus robinsi* | 2013 | GAM | 0.412 | 0.820 | 0.502 |
| *Bothus robinsi* | 2013 | GLM | 0.434 | 0.791 | 0.478 |
| *Bothus robinsi* | 2013 | RF | 0.532 | 0.704 | 0.402 |
| *Bothus robinsi* | 2013 | BRT | 0.373 | 0.891 | 0.651 |
| *Bothus robinsi* | 2014 | GAM | 0.444 | 0.724 | 0.395 |
| *Bothus robinsi* | 2014 | GLM | 0.452 | 0.724 | 0.316 |
| *Bothus robinsi* | 2014 | RF | 0.623 | 0.567 | 0.154 |
| *Bothus robinsi* | 2014 | BRT | 0.417 | 0.814 | 0.455 |
| *Bothus robinsi* | 2015 | GAM | 0.409 | 0.814 | 0.524 |
| *Bothus robinsi* | 2015 | GLM | 0.450 | 0.754 | 0.412 |
| *Bothus robinsi* | 2015 | RF | 0.525 | 0.700 | 0.420 |
| *Bothus robinsi* | 2015 | BRT | 0.386 | 0.860 | 0.575 |
| *Bothus robinsi* | 2016 | GAM | 0.471 | 0.684 | 0.271 |
| *Bothus robinsi* | 2016 | GLM | 0.478 | 0.672 | 0.326 |
| *Bothus robinsi* | 2016 | RF | 0.617 | 0.611 | 0.210 |
| *Bothus robinsi* | 2016 | BRT | 0.438 | 0.789 | 0.460 |
| *Bothus robinsi* | 2017 | GAM | 0.452 | 0.742 | 0.380 |
| *Bothus robinsi* | 2017 | GLM | 0.461 | 0.740 | 0.384 |
| *Bothus robinsi* | 2017 | RF | 0.649 | 0.578 | 0.163 |
| *Bothus robinsi* | 2017 | BRT | 0.434 | 0.791 | 0.464 |
| *Bothus robinsi* | 2018 | GAM | 0.462 | 0.708 | 0.338 |
| *Bothus robinsi* | 2018 | GLM | 0.465 | 0.718 | 0.363 |
| *Bothus robinsi* | 2018 | RF | 0.587 | 0.656 | 0.313 |
| *Bothus robinsi* | 2018 | BRT | 0.418 | 0.826 | 0.508 |
| *Bothus robinsi* | 2019 | GAM | 0.447 | 0.760 | 0.432 |
| *Bothus robinsi* | 2019 | GLM | 0.452 | 0.765 | 0.432 |
| *Bothus robinsi* | 2019 | RF | 0.563 | 0.683 | 0.364 |
| *Bothus robinsi* | 2019 | BRT | 0.421 | 0.826 | 0.541 |
| *Bothus robinsi* | 2020 | GAM | 0.406 | 0.800 | 0.475 |
| *Bothus robinsi* | 2020 | GLM | 0.410 | 0.802 | 0.588 |
| *Bothus robinsi* | 2020 | RF | 0.492 | 0.740 | 0.503 |
| *Bothus robinsi* | 2020 | BRT | 0.383 | 0.881 | 0.604 |
| *Bothus robinsi* | pooled | GAM | 0.456 | 0.710 | 0.314 |
| *Bothus robinsi* | pooled | GLM | 0.462 | 0.708 | 0.304 |
| *Bothus robinsi* | pooled | RF | 0.617 | 0.602 | 0.204 |
| *Bothus robinsi* | pooled | BRT | 0.449 | 0.737 | 0.346 |
| *Calamus proridens* | 2009 | GAM | 0.394 | 0.855 | 0.575 |
| *Calamus proridens* | 2009 | GLM | 0.412 | 0.832 | 0.528 |
| *Calamus proridens* | 2009 | RF | 0.435 | 0.811 | 0.619 |
| *Calamus proridens* | 2009 | BRT | 0.339 | 0.921 | 0.700 |
| *Calamus proridens* | 2010 | GAM | 0.388 | 0.859 | 0.604 |
| *Calamus proridens* | 2010 | GLM | 0.401 | 0.850 | 0.587 |
| *Calamus proridens* | 2010 | RF | 0.432 | 0.813 | 0.620 |
| *Calamus proridens* | 2010 | BRT | 0.351 | 0.909 | 0.644 |
| *Calamus proridens* | 2011 | GAM | 0.342 | 0.895 | 0.651 |
| *Calamus proridens* | 2011 | GLM | 0.371 | 0.888 | 0.620 |
| *Calamus proridens* | 2011 | RF | 0.456 | 0.763 | 0.533 |
| *Calamus proridens* | 2011 | BRT | 0.289 | 0.953 | 0.769 |
| *Calamus proridens* | 2012 | GAM | 0.398 | 0.848 | 0.584 |
| *Calamus proridens* | 2012 | GLM | 0.405 | 0.851 | 0.598 |
| *Calamus proridens* | 2012 | RF | 0.517 | 0.729 | 0.449 |
| *Calamus proridens* | 2012 | BRT | 0.363 | 0.898 | 0.665 |
| *Calamus proridens* | 2013 | GAM | 0.398 | 0.836 | 0.587 |
| *Calamus proridens* | 2013 | GLM | 0.416 | 0.827 | 0.533 |
| *Calamus proridens* | 2013 | RF | 0.541 | 0.690 | 0.381 |
| *Calamus proridens* | 2013 | BRT | 0.372 | 0.880 | 0.581 |
| *Calamus proridens* | 2014 | GAM | 0.416 | 0.811 | 0.485 |
| *Calamus proridens* | 2014 | GLM | 0.434 | 0.786 | 0.498 |
| *Calamus proridens* | 2014 | RF | 0.489 | 0.741 | 0.492 |
| *Calamus proridens* | 2014 | BRT | 0.379 | 0.870 | 0.563 |
| *Calamus proridens* | 2015 | GAM | 0.379 | 0.866 | 0.589 |
| *Calamus proridens* | 2015 | GLM | 0.425 | 0.807 | 0.556 |
| *Calamus proridens* | 2015 | RF | 0.492 | 0.757 | 0.496 |
| *Calamus proridens* | 2015 | BRT | 0.361 | 0.886 | 0.637 |
| *Calamus proridens* | 2016 | GAM | 0.406 | 0.810 | 0.447 |
| *Calamus proridens* | 2016 | GLM | 0.419 | 0.792 | 0.444 |
| *Calamus proridens* | 2016 | RF | 0.560 | 0.637 | 0.299 |
| *Calamus proridens* | 2016 | BRT | 0.378 | 0.865 | 0.545 |
| *Calamus proridens* | 2017 | GAM | 0.419 | 0.805 | 0.541 |
| *Calamus proridens* | 2017 | GLM | 0.429 | 0.795 | 0.487 |
| *Calamus proridens* | 2017 | RF | 0.536 | 0.703 | 0.398 |
| *Calamus proridens* | 2017 | BRT | 0.388 | 0.860 | 0.590 |
| *Calamus proridens* | 2018 | GAM | 0.418 | 0.790 | 0.477 |
| *Calamus proridens* | 2018 | GLM | 0.434 | 0.766 | 0.416 |
| *Calamus proridens* | 2018 | RF | 0.592 | 0.621 | 0.241 |
| *Calamus proridens* | 2018 | BRT | 0.393 | 0.845 | 0.546 |
| *Calamus proridens* | 2019 | GAM | 0.429 | 0.746 | 0.379 |
| *Calamus proridens* | 2019 | GLM | 0.436 | 0.733 | 0.389 |
| *Calamus proridens* | 2019 | RF | 0.560 | 0.626 | 0.275 |
| *Calamus proridens* | 2019 | BRT | 0.392 | 0.846 | 0.494 |
| *Calamus proridens* | 2020 | GAM | 0.380 | 0.866 | 0.633 |
| *Calamus proridens* | 2020 | GLM | 0.396 | 0.862 | 0.669 |
| *Calamus proridens* | 2020 | RF | 0.477 | 0.772 | 0.536 |
| *Calamus proridens* | 2020 | BRT | 0.350 | 0.914 | 0.745 |
| *Calamus proridens* | pooled | GAM | 0.425 | 0.790 | 0.435 |
| *Calamus proridens* | pooled | GLM | 0.437 | 0.776 | 0.425 |
| *Calamus proridens* | pooled | RF | 0.540 | 0.692 | 0.401 |
| *Calamus proridens* | pooled | BRT | 0.419 | 0.805 | 0.459 |
| *Diplectrum formosum* | 2009 | GAM | 0.277 | 0.878 | 0.722 |
| *Diplectrum formosum* | 2009 | GLM | 0.286 | 0.891 | 0.709 |
| *Diplectrum formosum* | 2009 | RF | 0.306 | 0.809 | 0.780 |
| *Diplectrum formosum* | 2009 | BRT | 0.260 | 0.940 | 0.811 |
| *Diplectrum formosum* | 2010 | GAM | 0.384 | 0.832 | 0.501 |
| *Diplectrum formosum* | 2010 | GLM | 0.387 | 0.836 | 0.564 |
| *Diplectrum formosum* | 2010 | RF | 0.520 | 0.678 | 0.390 |
| *Diplectrum formosum* | 2010 | BRT | 0.362 | 0.875 | 0.628 |
| *Diplectrum formosum* | 2011 | GAM | 0.316 | 0.891 | 0.634 |
| *Diplectrum formosum* | 2011 | GLM | 0.325 | 0.897 | 0.628 |
| *Diplectrum formosum* | 2011 | RF | 0.446 | 0.703 | 0.469 |
| *Diplectrum formosum* | 2011 | BRT | 0.284 | 0.943 | 0.660 |
| *Diplectrum formosum* | 2012 | GAM | 0.361 | 0.753 | 0.566 |
| *Diplectrum formosum* | 2012 | GLM | 0.368 | 0.754 | 0.564 |
| *Diplectrum formosum* | 2012 | RF | 0.455 | 0.663 | 0.430 |
| *Diplectrum formosum* | 2012 | BRT | 0.337 | 0.861 | 0.437 |
| *Diplectrum formosum* | 2013 | GAM | 0.292 | 0.889 | 0.487 |
| *Diplectrum formosum* | 2013 | GLM | 0.303 | 0.892 | 0.549 |
| *Diplectrum formosum* | 2013 | RF | 0.349 | 0.796 | 0.656 |
| *Diplectrum formosum* | 2013 | BRT | 0.259 | 0.944 | 0.732 |
| *Diplectrum formosum* | 2014 | GAM | 0.336 | 0.857 | 0.481 |
| *Diplectrum formosum* | 2014 | GLM | 0.346 | 0.849 | 0.478 |
| *Diplectrum formosum* | 2014 | RF | 0.437 | 0.721 | 0.486 |
| *Diplectrum formosum* | 2014 | BRT | 0.319 | 0.902 | 0.590 |
| *Diplectrum formosum* | 2015 | GAM | 0.319 | 0.857 | 0.438 |
| *Diplectrum formosum* | 2015 | GLM | 0.328 | 0.857 | 0.492 |
| *Diplectrum formosum* | 2015 | RF | 0.373 | 0.766 | 0.622 |
| *Diplectrum formosum* | 2015 | BRT | 0.292 | 0.913 | 0.670 |
| *Diplectrum formosum* | 2016 | GAM | 0.378 | 0.783 | 0.361 |
| *Diplectrum formosum* | 2016 | GLM | 0.381 | 0.755 | 0.448 |
| *Diplectrum formosum* | 2016 | RF | 0.475 | 0.637 | 0.351 |
| *Diplectrum formosum* | 2016 | BRT | 0.354 | 0.861 | 0.521 |
| *Diplectrum formosum* | 2017 | GAM | 0.244 | 0.913 | 0.335 |
| *Diplectrum formosum* | 2017 | GLM | 0.265 | 0.894 | 0.594 |
| *Diplectrum formosum* | 2017 | RF | 0.322 | 0.730 | 0.576 |
| *Diplectrum formosum* | 2017 | BRT | 0.217 | 0.952 | 0.684 |
| *Diplectrum formosum* | 2018 | GAM | 0.321 | 0.798 | 0.542 |
| *Diplectrum formosum* | 2018 | GLM | 0.336 | 0.781 | 0.543 |
| *Diplectrum formosum* | 2018 | RF | 0.384 | 0.692 | 0.522 |
| *Diplectrum formosum* | 2018 | BRT | 0.298 | 0.888 | 0.450 |
| *Diplectrum formosum* | 2019 | GAM | 0.307 | 0.784 | 0.540 |
| *Diplectrum formosum* | 2019 | GLM | 0.311 | 0.777 | 0.631 |
| *Diplectrum formosum* | 2019 | RF | 0.365 | 0.686 | 0.569 |
| *Diplectrum formosum* | 2019 | BRT | 0.290 | 0.882 | 0.541 |
| *Diplectrum formosum* | 2020 | GAM | 0.321 | 0.887 | 0.561 |
| *Diplectrum formosum* | 2020 | GLM | 0.322 | 0.892 | 0.563 |
| *Diplectrum formosum* | 2020 | RF | 0.554 | 0.532 | 0.072 |
| *Diplectrum formosum* | 2020 | BRT | 0.293 | 0.939 | 0.673 |
| *Diplectrum formosum* | pooled | GAM | 0.335 | 0.819 | 0.461 |
| *Diplectrum formosum* | pooled | GLM | 0.344 | 0.815 | 0.384 |
| *Diplectrum formosum* | pooled | RF | 0.412 | 0.700 | 0.524 |
| *Diplectrum formosum* | pooled | BRT | 0.331 | 0.832 | 0.406 |
| *Haemulon aurolineatum* | 2009 | GAM | 0.404 | 0.815 | 0.612 |
| *Haemulon aurolineatum* | 2009 | GLM | 0.423 | 0.779 | 0.621 |
| *Haemulon aurolineatum* | 2009 | RF | 0.558 | 0.679 | 0.377 |
| *Haemulon aurolineatum* | 2009 | BRT | 0.381 | 0.870 | 0.625 |
| *Haemulon aurolineatum* | 2010 | GAM | 0.451 | 0.737 | 0.511 |
| *Haemulon aurolineatum* | 2010 | GLM | 0.454 | 0.740 | 0.391 |
| *Haemulon aurolineatum* | 2010 | RF | 0.578 | 0.668 | 0.341 |
| *Haemulon aurolineatum* | 2010 | BRT | 0.416 | 0.825 | 0.514 |
| *Haemulon aurolineatum* | 2011 | GAM | 0.437 | 0.778 | 0.478 |
| *Haemulon aurolineatum* | 2011 | GLM | 0.437 | 0.780 | 0.467 |
| *Haemulon aurolineatum* | 2011 | RF | 0.599 | 0.636 | 0.283 |
| *Haemulon aurolineatum* | 2011 | BRT | 0.397 | 0.857 | 0.611 |
| *Haemulon aurolineatum* | 2012 | GAM | 0.408 | 0.814 | 0.619 |
| *Haemulon aurolineatum* | 2012 | GLM | 0.410 | 0.817 | 0.556 |
| *Haemulon aurolineatum* | 2012 | RF | 0.546 | 0.688 | 0.396 |
| *Haemulon aurolineatum* | 2012 | BRT | 0.377 | 0.879 | 0.603 |
| *Haemulon aurolineatum* | 2013 | GAM | 0.430 | 0.778 | 0.503 |
| *Haemulon aurolineatum* | 2013 | GLM | 0.444 | 0.763 | 0.471 |
| *Haemulon aurolineatum* | 2013 | RF | 0.526 | 0.717 | 0.446 |
| *Haemulon aurolineatum* | 2013 | BRT | 0.395 | 0.856 | 0.559 |
| *Haemulon aurolineatum* | 2014 | GAM | 0.453 | 0.743 | 0.394 |
| *Haemulon aurolineatum* | 2014 | GLM | 0.454 | 0.740 | 0.377 |
| *Haemulon aurolineatum* | 2014 | RF | 0.583 | 0.656 | 0.339 |
| *Haemulon aurolineatum* | 2014 | BRT | 0.417 | 0.823 | 0.479 |
| *Haemulon aurolineatum* | 2015 | GAM | 0.420 | 0.810 | 0.552 |
| *Haemulon aurolineatum* | 2015 | GLM | 0.442 | 0.785 | 0.538 |
| *Haemulon aurolineatum* | 2015 | RF | 0.531 | 0.717 | 0.423 |
| *Haemulon aurolineatum* | 2015 | BRT | 0.388 | 0.870 | 0.625 |
| *Haemulon aurolineatum* | 2016 | GAM | 0.445 | 0.746 | 0.404 |
| *Haemulon aurolineatum* | 2016 | GLM | 0.451 | 0.730 | 0.386 |
| *Haemulon aurolineatum* | 2016 | RF | 0.599 | 0.621 | 0.260 |
| *Haemulon aurolineatum* | 2016 | BRT | 0.413 | 0.826 | 0.491 |
| *Haemulon aurolineatum* | 2017 | GAM | 0.430 | 0.793 | 0.447 |
| *Haemulon aurolineatum* | 2017 | GLM | 0.432 | 0.792 | 0.443 |
| *Haemulon aurolineatum* | 2017 | RF | 0.578 | 0.664 | 0.340 |
| *Haemulon aurolineatum* | 2017 | BRT | 0.400 | 0.852 | 0.572 |
| *Haemulon aurolineatum* | 2018 | GAM | 0.431 | 0.763 | 0.536 |
| *Haemulon aurolineatum* | 2018 | GLM | 0.446 | 0.750 | 0.587 |
| *Haemulon aurolineatum* | 2018 | RF | 0.576 | 0.670 | 0.340 |
| *Haemulon aurolineatum* | 2018 | BRT | 0.406 | 0.839 | 0.549 |
| *Haemulon aurolineatum* | 2019 | GAM | 0.448 | 0.755 | 0.438 |
| *Haemulon aurolineatum* | 2019 | GLM | 0.446 | 0.756 | 0.447 |
| *Haemulon aurolineatum* | 2019 | RF | 0.599 | 0.640 | 0.278 |
| *Haemulon aurolineatum* | 2019 | BRT | 0.422 | 0.814 | 0.509 |
| *Haemulon aurolineatum* | 2020 | GAM | 0.432 | 0.770 | 0.598 |
| *Haemulon aurolineatum* | 2020 | GLM | 0.435 | 0.765 | 0.609 |
| *Haemulon aurolineatum* | 2020 | RF | 0.620 | 0.609 | 0.218 |
| *Haemulon aurolineatum* | 2020 | BRT | 0.378 | 0.882 | 0.649 |
| *Haemulon aurolineatum* | pooled | GAM | 0.442 | 0.762 | 0.421 |
| *Haemulon aurolineatum* | pooled | GLM | 0.448 | 0.753 | 0.423 |
| *Haemulon aurolineatum* | pooled | RF | 0.574 | 0.671 | 0.346 |
| *Haemulon aurolineatum* | pooled | BRT | 0.436 | 0.777 | 0.440 |
| *Lagodon rhomboides* | 2009 | GAM | 0.468 | 0.700 | 0.310 |
| *Lagodon rhomboides* | 2009 | GLM | 0.473 | 0.678 | 0.276 |
| *Lagodon rhomboides* | 2009 | RF | 0.614 | 0.621 | 0.240 |
| *Lagodon rhomboides* | 2009 | BRT | 0.434 | 0.791 | 0.438 |
| *Lagodon rhomboides* | 2010 | GAM | 0.356 | 0.840 | 0.553 |
| *Lagodon rhomboides* | 2010 | GLM | 0.376 | 0.822 | 0.553 |
| *Lagodon rhomboides* | 2010 | RF | 0.453 | 0.722 | 0.476 |
| *Lagodon rhomboides* | 2010 | BRT | 0.343 | 0.850 | 0.634 |
| *Lagodon rhomboides* | 2011 | GAM | 0.425 | 0.785 | 0.496 |
| *Lagodon rhomboides* | 2011 | GLM | 0.437 | 0.770 | 0.458 |
| *Lagodon rhomboides* | 2011 | RF | 0.544 | 0.695 | 0.382 |
| *Lagodon rhomboides* | 2011 | BRT | 0.394 | 0.859 | 0.546 |
| *Lagodon rhomboides* | 2012 | GAM | 0.400 | 0.842 | 0.593 |
| *Lagodon rhomboides* | 2012 | GLM | 0.406 | 0.838 | 0.570 |
| *Lagodon rhomboides* | 2012 | RF | 0.504 | 0.743 | 0.479 |
| *Lagodon rhomboides* | 2012 | BRT | 0.369 | 0.887 | 0.627 |
| *Lagodon rhomboides* | 2013 | GAM | 0.347 | 0.874 | 0.570 |
| *Lagodon rhomboides* | 2013 | GLM | 0.359 | 0.867 | 0.606 |
| *Lagodon rhomboides* | 2013 | RF | 0.387 | 0.806 | 0.670 |
| *Lagodon rhomboides* | 2013 | BRT | 0.314 | 0.917 | 0.782 |
| *Lagodon rhomboides* | 2014 | GAM | 0.403 | 0.794 | 0.559 |
| *Lagodon rhomboides* | 2014 | GLM | 0.416 | 0.757 | 0.547 |
| *Lagodon rhomboides* | 2014 | RF | 0.501 | 0.689 | 0.438 |
| *Lagodon rhomboides* | 2014 | BRT | 0.381 | 0.852 | 0.547 |
| *Lagodon rhomboides* | 2015 | GAM | 0.365 | 0.848 | 0.470 |
| *Lagodon rhomboides* | 2015 | GLM | 0.376 | 0.833 | 0.470 |
| *Lagodon rhomboides* | 2015 | RF | 0.451 | 0.726 | 0.470 |
| *Lagodon rhomboides* | 2015 | BRT | 0.331 | 0.903 | 0.574 |
| *Lagodon rhomboides* | 2016 | GAM | 0.315 | 0.884 | 0.429 |
| *Lagodon rhomboides* | 2016 | GLM | 0.324 | 0.874 | 0.405 |
| *Lagodon rhomboides* | 2016 | RF | 0.454 | 0.619 | 0.274 |
| *Lagodon rhomboides* | 2016 | BRT | 0.296 | 0.917 | 0.471 |
| *Lagodon rhomboides* | 2017 | GAM | 0.380 | 0.849 | 0.579 |
| *Lagodon rhomboides* | 2017 | GLM | 0.385 | 0.849 | 0.573 |
| *Lagodon rhomboides* | 2017 | RF | 0.532 | 0.685 | 0.372 |
| *Lagodon rhomboides* | 2017 | BRT | 0.358 | 0.890 | 0.604 |
| *Lagodon rhomboides* | 2018 | GAM | 0.332 | 0.906 | 0.668 |
| *Lagodon rhomboides* | 2018 | GLM | 0.353 | 0.888 | 0.653 |
| *Lagodon rhomboides* | 2018 | RF | 0.429 | 0.778 | 0.543 |
| *Lagodon rhomboides* | 2018 | BRT | 0.310 | 0.932 | 0.735 |
| *Lagodon rhomboides* | 2019 | GAM | 0.369 | 0.857 | 0.529 |
| *Lagodon rhomboides* | 2019 | GLM | 0.396 | 0.814 | 0.490 |
| *Lagodon rhomboides* | 2019 | RF | 0.486 | 0.707 | 0.454 |
| *Lagodon rhomboides* | 2019 | BRT | 0.358 | 0.874 | 0.629 |
| *Lagodon rhomboides* | 2020 | GAM | 0.390 | 0.855 | 0.591 |
| *Lagodon rhomboides* | 2020 | GLM | 0.388 | 0.846 | 0.617 |
| *Lagodon rhomboides* | 2020 | RF | 0.506 | 0.728 | 0.478 |
| *Lagodon rhomboides* | 2020 | BRT | 0.314 | 0.944 | 0.839 |
| *Lagodon rhomboides* | pooled | GAM | 0.398 | 0.803 | 0.435 |
| *Lagodon rhomboides* | pooled | GLM | 0.404 | 0.792 | 0.428 |
| *Lagodon rhomboides* | pooled | RF | 0.505 | 0.694 | 0.415 |
| *Lagodon rhomboides* | pooled | BRT | 0.392 | 0.821 | 0.455 |
| *Lutjanus synagris* | 2009 | GAM | 0.361 | 0.898 | 0.647 |
| *Lutjanus synagris* | 2009 | GLM | 0.437 | 0.839 | 0.536 |
| *Lutjanus synagris* | 2009 | RF | 0.440 | 0.804 | 0.613 |
| *Lutjanus synagris* | 2009 | BRT | 0.349 | 0.916 | 0.664 |
| *Lutjanus synagris* | 2010 | GAM | 0.374 | 0.863 | 0.523 |
| *Lutjanus synagris* | 2010 | GLM | 0.405 | 0.820 | 0.437 |
| *Lutjanus synagris* | 2010 | RF | 0.482 | 0.729 | 0.482 |
| *Lutjanus synagris* | 2010 | BRT | 0.353 | 0.895 | 0.612 |
| *Lutjanus synagris* | 2011 | GAM | 0.336 | 0.918 | 0.653 |
| *Lutjanus synagris* | 2011 | GLM | 0.402 | 0.859 | 0.627 |
| *Lutjanus synagris* | 2011 | RF | 0.457 | 0.772 | 0.530 |
| *Lutjanus synagris* | 2011 | BRT | 0.324 | 0.928 | 0.679 |
| *Lutjanus synagris* | 2012 | GAM | 0.398 | 0.841 | 0.590 |
| *Lutjanus synagris* | 2012 | GLM | 0.424 | 0.828 | 0.569 |
| *Lutjanus synagris* | 2012 | RF | 0.512 | 0.739 | 0.462 |
| *Lutjanus synagris* | 2012 | BRT | 0.363 | 0.899 | 0.666 |
| *Lutjanus synagris* | 2013 | GAM | 0.386 | 0.863 | 0.618 |
| *Lutjanus synagris* | 2013 | GLM | 0.401 | 0.857 | 0.618 |
| *Lutjanus synagris* | 2013 | RF | 0.485 | 0.755 | 0.512 |
| *Lutjanus synagris* | 2013 | BRT | 0.325 | 0.932 | 0.743 |
| *Lutjanus synagris* | 2014 | GAM | 0.386 | 0.868 | 0.565 |
| *Lutjanus synagris* | 2014 | GLM | 0.405 | 0.850 | 0.544 |
| *Lutjanus synagris* | 2014 | RF | 0.476 | 0.770 | 0.538 |
| *Lutjanus synagris* | 2014 | BRT | 0.354 | 0.906 | 0.673 |
| *Lutjanus synagris* | 2015 | GAM | 0.355 | 0.902 | 0.657 |
| *Lutjanus synagris* | 2015 | GLM | 0.421 | 0.824 | 0.555 |
| *Lutjanus synagris* | 2015 | RF | 0.447 | 0.794 | 0.586 |
| *Lutjanus synagris* | 2015 | BRT | 0.336 | 0.923 | 0.697 |
| *Lutjanus synagris* | 2016 | GAM | 0.361 | 0.886 | 0.584 |
| *Lutjanus synagris* | 2016 | GLM | 0.415 | 0.817 | 0.505 |
| *Lutjanus synagris* | 2016 | RF | 0.496 | 0.728 | 0.487 |
| *Lutjanus synagris* | 2016 | BRT | 0.343 | 0.907 | 0.654 |
| *Lutjanus synagris* | 2017 | GAM | 0.334 | 0.923 | 0.728 |
| *Lutjanus synagris* | 2017 | GLM | 0.382 | 0.883 | 0.675 |
| *Lutjanus synagris* | 2017 | RF | 0.470 | 0.776 | 0.545 |
| *Lutjanus synagris* | 2017 | BRT | 0.317 | 0.938 | 0.760 |
| *Lutjanus synagris* | 2018 | GAM | 0.368 | 0.887 | 0.663 |
| *Lutjanus synagris* | 2018 | GLM | 0.408 | 0.851 | 0.573 |
| *Lutjanus synagris* | 2018 | RF | 0.459 | 0.788 | 0.568 |
| *Lutjanus synagris* | 2018 | BRT | 0.342 | 0.924 | 0.711 |
| *Lutjanus synagris* | 2019 | GAM | 0.358 | 0.899 | 0.625 |
| *Lutjanus synagris* | 2019 | GLM | 0.409 | 0.845 | 0.536 |
| *Lutjanus synagris* | 2019 | RF | 0.459 | 0.782 | 0.559 |
| *Lutjanus synagris* | 2019 | BRT | 0.333 | 0.925 | 0.667 |
| *Lutjanus synagris* | 2020 | GAM | 0.298 | 0.939 | 0.806 |
| *Lutjanus synagris* | 2020 | GLM | 0.337 | 0.939 | 0.770 |
| *Lutjanus synagris* | 2020 | RF | 0.403 | 0.836 | 0.669 |
| *Lutjanus synagris* | 2020 | BRT | 0.236 | 0.977 | 0.877 |
| *Lutjanus synagris* | pooled | GAM | 0.392 | 0.855 | 0.528 |
| *Lutjanus synagris* | pooled | GLM | 0.423 | 0.816 | 0.510 |
| *Lutjanus synagris* | pooled | RF | 0.485 | 0.757 | 0.510 |
| *Lutjanus synagris* | pooled | BRT | 0.386 | 0.865 | 0.558 |
| *Prionotus roseus* | 2009 | GAM | 0.412 | 0.816 | 0.481 |
| *Prionotus roseus* | 2009 | GLM | 0.416 | 0.812 | 0.455 |
| *Prionotus roseus* | 2009 | RF | 0.558 | 0.668 | 0.339 |
| *Prionotus roseus* | 2009 | BRT | 0.376 | 0.877 | 0.569 |
| *Prionotus roseus* | 2010 | GAM | 0.423 | 0.765 | 0.385 |
| *Prionotus roseus* | 2010 | GLM | 0.428 | 0.754 | 0.390 |
| *Prionotus roseus* | 2010 | RF | 0.564 | 0.624 | 0.272 |
| *Prionotus roseus* | 2010 | BRT | 0.401 | 0.827 | 0.460 |
| *Prionotus roseus* | 2011 | GAM | 0.406 | 0.787 | 0.420 |
| *Prionotus roseus* | 2011 | GLM | 0.420 | 0.786 | 0.404 |
| *Prionotus roseus* | 2011 | RF | 0.585 | 0.562 | 0.130 |
| *Prionotus roseus* | 2011 | BRT | 0.371 | 0.855 | 0.453 |
| *Prionotus roseus* | 2012 | GAM | 0.421 | 0.736 | 0.321 |
| *Prionotus roseus* | 2012 | GLM | 0.420 | 0.740 | 0.319 |
| *Prionotus roseus* | 2012 | RF | 0.545 | 0.618 | 0.270 |
| *Prionotus roseus* | 2012 | BRT | 0.386 | 0.836 | 0.407 |
| *Prionotus roseus* | 2013 | GAM | 0.422 | 0.782 | 0.437 |
| *Prionotus roseus* | 2013 | GLM | 0.435 | 0.753 | 0.374 |
| *Prionotus roseus* | 2013 | RF | 0.605 | 0.585 | 0.184 |
| *Prionotus roseus* | 2013 | BRT | 0.401 | 0.836 | 0.477 |
| *Prionotus roseus* | 2014 | GAM | 0.418 | 0.767 | 0.403 |
| *Prionotus roseus* | 2014 | GLM | 0.420 | 0.768 | 0.378 |
| *Prionotus roseus* | 2014 | RF | 0.518 | 0.664 | 0.366 |
| *Prionotus roseus* | 2014 | BRT | 0.385 | 0.846 | 0.490 |
| *Prionotus roseus* | 2015 | GAM | 0.422 | 0.783 | 0.441 |
| *Prionotus roseus* | 2015 | GLM | 0.433 | 0.764 | 0.455 |
| *Prionotus roseus* | 2015 | RF | 0.559 | 0.649 | 0.311 |
| *Prionotus roseus* | 2015 | BRT | 0.398 | 0.846 | 0.502 |
| *Prionotus roseus* | 2016 | GAM | 0.444 | 0.731 | 0.310 |
| *Prionotus roseus* | 2016 | GLM | 0.450 | 0.718 | 0.336 |
| *Prionotus roseus* | 2016 | RF | 0.597 | 0.604 | 0.231 |
| *Prionotus roseus* | 2016 | BRT | 0.409 | 0.825 | 0.486 |
| *Prionotus roseus* | 2017 | GAM | 0.423 | 0.791 | 0.470 |
| *Prionotus roseus* | 2017 | GLM | 0.430 | 0.791 | 0.443 |
| *Prionotus roseus* | 2017 | RF | 0.544 | 0.680 | 0.367 |
| *Prionotus roseus* | 2017 | BRT | 0.384 | 0.865 | 0.582 |
| *Prionotus roseus* | 2018 | GAM | 0.410 | 0.827 | 0.507 |
| *Prionotus roseus* | 2018 | GLM | 0.417 | 0.825 | 0.536 |
| *Prionotus roseus* | 2018 | RF | 0.555 | 0.686 | 0.386 |
| *Prionotus roseus* | 2018 | BRT | 0.385 | 0.869 | 0.579 |
| *Prionotus roseus* | 2019 | GAM | 0.441 | 0.758 | 0.482 |
| *Prionotus roseus* | 2019 | GLM | 0.441 | 0.754 | 0.485 |
| *Prionotus roseus* | 2019 | RF | 0.589 | 0.655 | 0.314 |
| *Prionotus roseus* | 2019 | BRT | 0.417 | 0.821 | 0.551 |
| *Prionotus roseus* | 2020 | GAM | 0.405 | 0.805 | 0.615 |
| *Prionotus roseus* | 2020 | GLM | 0.402 | 0.810 | 0.607 |
| *Prionotus roseus* | 2020 | RF | 0.571 | 0.669 | 0.356 |
| *Prionotus roseus* | 2020 | BRT | 0.358 | 0.905 | 0.699 |
| *Prionotus roseus* | pooled | GAM | 0.443 | 0.734 | 0.343 |
| *Prionotus roseus* | pooled | GLM | 0.443 | 0.731 | 0.349 |
| *Prionotus roseus* | pooled | RF | 0.591 | 0.615 | 0.248 |
| *Prionotus roseus* | pooled | BRT | 0.437 | 0.749 | 0.343 |
| *Synodus foetens* | 2009 | GAM | 0.349 | 0.776 | 0.357 |
| *Synodus foetens* | 2009 | GLM | 0.351 | 0.776 | 0.362 |
| *Synodus foetens* | 2009 | RF | 0.401 | 0.690 | 0.544 |
| *Synodus foetens* | 2009 | BRT | 0.317 | 0.858 | 0.538 |
| *Synodus foetens* | 2010 | GAM | 0.370 | 0.817 | 0.466 |
| *Synodus foetens* | 2010 | GLM | 0.379 | 0.811 | 0.389 |
| *Synodus foetens* | 2010 | RF | 0.415 | 0.742 | 0.527 |
| *Synodus foetens* | 2010 | BRT | 0.331 | 0.903 | 0.602 |
| *Synodus foetens* | 2011 | GAM | 0.383 | 0.839 | 0.509 |
| *Synodus foetens* | 2011 | GLM | 0.380 | 0.850 | 0.517 |
| *Synodus foetens* | 2011 | RF | 0.485 | 0.710 | 0.425 |
| *Synodus foetens* | 2011 | BRT | 0.319 | 0.910 | 0.646 |
| *Synodus foetens* | 2012 | GAM | 0.425 | 0.746 | 0.369 |
| *Synodus foetens* | 2012 | GLM | 0.421 | 0.759 | 0.380 |
| *Synodus foetens* | 2012 | RF | 0.517 | 0.678 | 0.399 |
| *Synodus foetens* | 2012 | BRT | 0.386 | 0.835 | 0.574 |
| *Synodus foetens* | 2013 | GAM | 0.411 | 0.724 | 0.382 |
| *Synodus foetens* | 2013 | GLM | 0.413 | 0.726 | 0.371 |
| *Synodus foetens* | 2013 | RF | 0.529 | 0.616 | 0.315 |
| *Synodus foetens* | 2013 | BRT | 0.376 | 0.851 | 0.514 |
| *Synodus foetens* | 2014 | GAM | 0.415 | 0.772 | 0.376 |
| *Synodus foetens* | 2014 | GLM | 0.427 | 0.755 | 0.367 |
| *Synodus foetens* | 2014 | RF | 0.524 | 0.667 | 0.350 |
| *Synodus foetens* | 2014 | BRT | 0.392 | 0.828 | 0.504 |
| *Synodus foetens* | 2015 | GAM | 0.462 | 0.699 | 0.328 |
| *Synodus foetens* | 2015 | GLM | 0.463 | 0.706 | 0.342 |
| *Synodus foetens* | 2015 | RF | 0.633 | 0.586 | 0.172 |
| *Synodus foetens* | 2015 | BRT | 0.412 | 0.847 | 0.550 |
| *Synodus foetens* | 2016 | GAM | 0.443 | 0.710 | 0.312 |
| *Synodus foetens* | 2016 | GLM | 0.444 | 0.709 | 0.298 |
| *Synodus foetens* | 2016 | RF | 0.604 | 0.577 | 0.183 |
| *Synodus foetens* | 2016 | BRT | 0.411 | 0.810 | 0.474 |
| *Synodus foetens* | 2017 | GAM | 0.375 | 0.806 | 0.388 |
| *Synodus foetens* | 2017 | GLM | 0.381 | 0.803 | 0.488 |
| *Synodus foetens* | 2017 | RF | 0.505 | 0.642 | 0.315 |
| *Synodus foetens* | 2017 | BRT | 0.340 | 0.881 | 0.527 |
| *Synodus foetens* | 2018 | GAM | 0.400 | 0.704 | 0.473 |
| *Synodus foetens* | 2018 | GLM | 0.400 | 0.719 | 0.428 |
| *Synodus foetens* | 2018 | RF | 0.504 | 0.607 | 0.287 |
| *Synodus foetens* | 2018 | BRT | 0.368 | 0.855 | 0.453 |
| *Synodus foetens* | 2019 | GAM | 0.350 | 0.806 | 0.413 |
| *Synodus foetens* | 2019 | GLM | 0.358 | 0.793 | 0.505 |
| *Synodus foetens* | 2019 | RF | 0.414 | 0.708 | 0.526 |
| *Synodus foetens* | 2019 | BRT | 0.317 | 0.878 | 0.689 |
| *Synodus foetens* | 2020 | GAM | 0.383 | 0.802 | 0.711 |
| *Synodus foetens* | 2020 | GLM | 0.403 | 0.779 | 0.690 |
| *Synodus foetens* | 2020 | RF | 0.501 | 0.680 | 0.419 |
| *Synodus foetens* | 2020 | BRT | 0.346 | 0.898 | 0.621 |
| *Synodus foetens* | pooled | GAM | 0.413 | 0.723 | 0.394 |
| *Synodus foetens* | pooled | GLM | 0.418 | 0.716 | 0.440 |
| *Synodus foetens* | pooled | RF | 0.506 | 0.635 | 0.359 |
| *Synodus foetens* | pooled | BRT | 0.407 | 0.750 | 0.348 |
| *Trachinocephalus myops* | 2009 | GAM | 0.459 | 0.726 | 0.386 |
| *Trachinocephalus myops* | 2009 | GLM | 0.458 | 0.730 | 0.375 |
| *Trachinocephalus myops* | 2009 | RF | 0.573 | 0.669 | 0.344 |
| *Trachinocephalus myops* | 2009 | BRT | 0.420 | 0.818 | 0.518 |
| *Trachinocephalus myops* | 2010 | GAM | 0.482 | 0.661 | 0.260 |
| *Trachinocephalus myops* | 2010 | GLM | 0.479 | 0.667 | 0.239 |
| *Trachinocephalus myops* | 2010 | RF | 0.610 | 0.627 | 0.258 |
| *Trachinocephalus myops* | 2010 | BRT | 0.448 | 0.757 | 0.385 |
| *Trachinocephalus myops* | 2011 | GAM | 0.459 | 0.720 | 0.356 |
| *Trachinocephalus myops* | 2011 | GLM | 0.458 | 0.725 | 0.353 |
| *Trachinocephalus myops* | 2011 | RF | 0.596 | 0.630 | 0.288 |
| *Trachinocephalus myops* | 2011 | BRT | 0.402 | 0.842 | 0.563 |
| *Trachinocephalus myops* | 2012 | GAM | 0.452 | 0.705 | 0.343 |
| *Trachinocephalus myops* | 2012 | GLM | 0.451 | 0.715 | 0.363 |
| *Trachinocephalus myops* | 2012 | RF | 0.628 | 0.566 | 0.160 |
| *Trachinocephalus myops* | 2012 | BRT | 0.424 | 0.790 | 0.459 |
| *Trachinocephalus myops* | 2013 | GAM | 0.445 | 0.741 | 0.377 |
| *Trachinocephalus myops* | 2013 | GLM | 0.448 | 0.725 | 0.437 |
| *Trachinocephalus myops* | 2013 | RF | 0.576 | 0.648 | 0.319 |
| *Trachinocephalus myops* | 2013 | BRT | 0.412 | 0.834 | 0.542 |
| *Trachinocephalus myops* | 2014 | GAM | 0.440 | 0.775 | 0.449 |
| *Trachinocephalus myops* | 2014 | GLM | 0.448 | 0.758 | 0.375 |
| *Trachinocephalus myops* | 2014 | RF | 0.549 | 0.696 | 0.404 |
| *Trachinocephalus myops* | 2014 | BRT | 0.398 | 0.859 | 0.563 |
| *Trachinocephalus myops* | 2015 | GAM | 0.473 | 0.679 | 0.267 |
| *Trachinocephalus myops* | 2015 | GLM | 0.470 | 0.690 | 0.300 |
| *Trachinocephalus myops* | 2015 | RF | 0.599 | 0.638 | 0.268 |
| *Trachinocephalus myops* | 2015 | BRT | 0.450 | 0.754 | 0.419 |
| *Trachinocephalus myops* | 2016 | GAM | 0.459 | 0.717 | 0.392 |
| *Trachinocephalus myops* | 2016 | GLM | 0.459 | 0.711 | 0.451 |
| *Trachinocephalus myops* | 2016 | RF | 0.616 | 0.621 | 0.240 |
| *Trachinocephalus myops* | 2016 | BRT | 0.425 | 0.819 | 0.531 |
| *Trachinocephalus myops* | 2017 | GAM | 0.424 | 0.773 | 0.446 |
| *Trachinocephalus myops* | 2017 | GLM | 0.424 | 0.773 | 0.480 |
| *Trachinocephalus myops* | 2017 | RF | 0.571 | 0.631 | 0.302 |
| *Trachinocephalus myops* | 2017 | BRT | 0.402 | 0.826 | 0.487 |
| *Trachinocephalus myops* | 2018 | GAM | 0.377 | 0.866 | 0.599 |
| *Trachinocephalus myops* | 2018 | GLM | 0.383 | 0.865 | 0.604 |
| *Trachinocephalus myops* | 2018 | RF | 0.526 | 0.712 | 0.428 |
| *Trachinocephalus myops* | 2018 | BRT | 0.365 | 0.892 | 0.678 |
| *Trachinocephalus myops* | 2019 | GAM | 0.422 | 0.785 | 0.423 |
| *Trachinocephalus myops* | 2019 | GLM | 0.420 | 0.787 | 0.433 |
| *Trachinocephalus myops* | 2019 | RF | 0.555 | 0.666 | 0.374 |
| *Trachinocephalus myops* | 2019 | BRT | 0.395 | 0.842 | 0.527 |
| *Trachinocephalus myops* | 2020 | GAM | 0.406 | 0.745 | 0.670 |
| *Trachinocephalus myops* | 2020 | GLM | 0.405 | 0.773 | 0.363 |
| *Trachinocephalus myops* | 2020 | RF | 0.516 | 0.639 | 0.339 |
| *Trachinocephalus myops* | 2020 | BRT | 0.373 | 0.864 | 0.496 |
| *Trachinocephalus myops* | pooled | GAM | 0.462 | 0.720 | 0.321 |
| *Trachinocephalus myops* | pooled | GLM | 0.461 | 0.722 | 0.321 |
| *Trachinocephalus myops* | pooled | RF | 0.586 | 0.656 | 0.318 |
| *Trachinocephalus myops* | pooled | BRT | 0.456 | 0.734 | 0.351 |


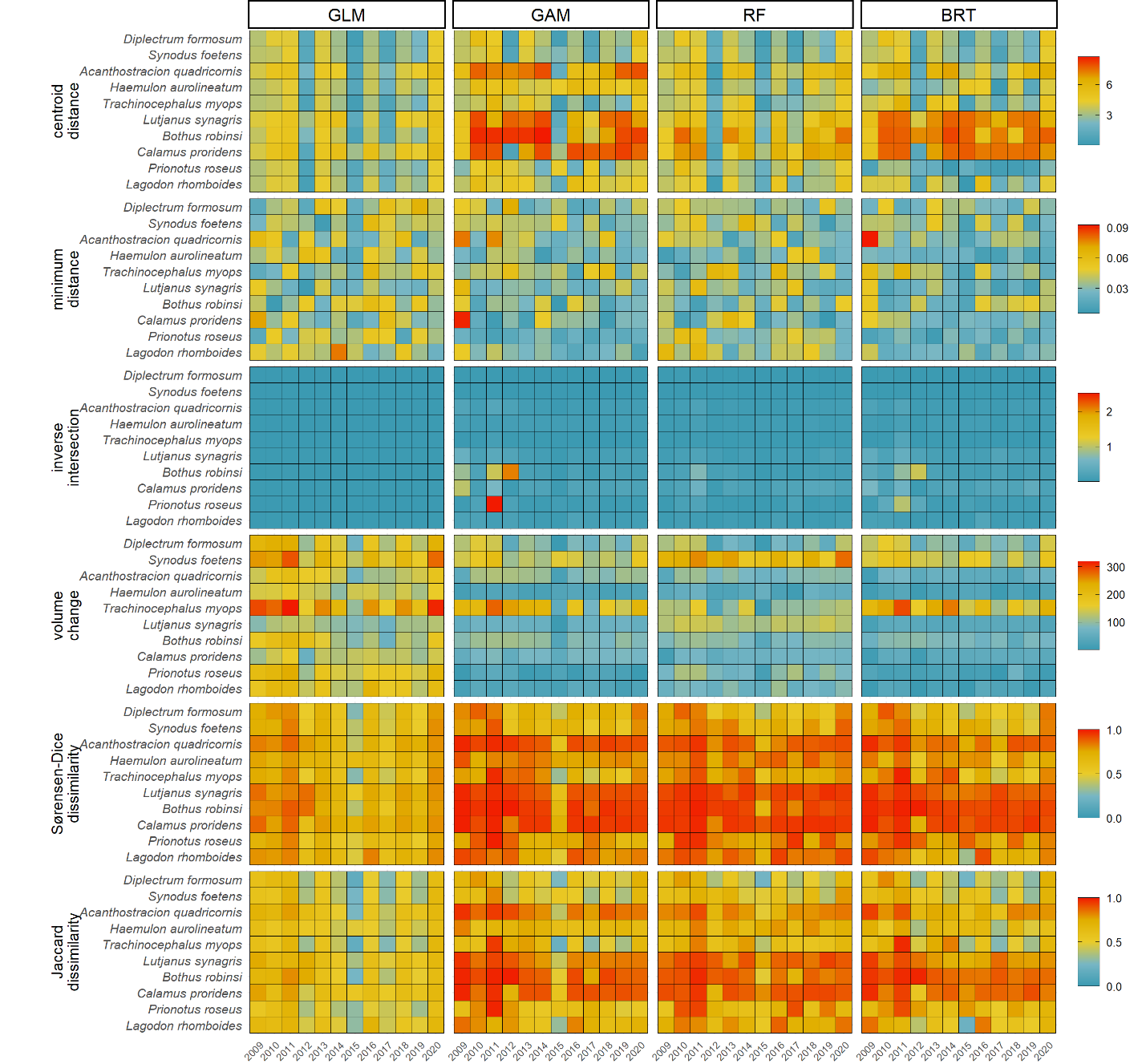


Figure S3. Hypervolume niche metrics for each ecological niche modeling (ENM) algorithm [Generalized Linear Model (GLM), Generalized Additive Model (GAM), Random Forest (RF), and Boosted Regression Trees (BRT)], species, and year. Colors intensity is proportional to the metric value (high: red; low: blue).

Table S2. Linear mixed models (LMMs) estimates, ANOVA p-value from likelihood ratio test (LRT), and marginal R^2^.

| **LMM** | **metric** | **estimate** | **LRT p-value** | **marginal R^2^** |
| --- | --- | --- | --- | --- |
| abundance | volume | -3.801 | 0.019 | 0.005 |
| frequency | volume | -10.933 | 0.001 | 0.037 |
| red tide | volume | 6.175 | <0.001 | 0.012 |
| sampling effort | volume | -12.460 | <0.001 | 0.049 |
| algorithm | volume | -67.232 | <0.001 | 0.427 |
| SSS | volume | -9.207 | <0.001 | 0.027 |
| SST | volume | -9.788 | <0.001 | 0.028 |
| abundance | distance | -0.060 | 0.148 | 0.001 |
| frequency | distance | -0.313 | 0.192 | 0.024 |
| red tide | distance | 0.256 | 0.004 | 0.016 |
| sampling effort | distance | -0.564 | <0.001 | 0.076 |
| algorithm | distance | 1.515 | <0.001 | 0.072 |
| SSS | distance | -0.670 | <0.001 | 0.106 |
| SST | distance | 0.067 | 0.108 | 0.001 |
| abundance | dissimilarity | 0.010 | 0.011 | 0.004 |
| frequency | dissimilarity | -0.038 | 0.131 | 0.070 |
| red tide | dissimilarity | 0.024 | <0.001 | 0.026 |
| sampling effort | dissimilarity | -0.043 | <0.001 | 0.084 |
| algorithm | dissimilarity | 0.144 | <0.001 | 0.143 |
| SSS | dissimilarity | -0.037 | <0.001 | 0.062 |
| SST | dissimilarity | -0.016 | 0.107 | 0.011 |
